# Too Cute for Words: Cuteness Evokes the Heartwarming Emotion of Kama Muta

**DOI:** 10.3389/fpsyg.2019.00387

**Published:** 2019-03-01

**Authors:** Kamilla Knutsen Steinnes, Johanna Katarina Blomster, Beate Seibt, Janis H. Zickfeld, Alan Page Fiske

**Affiliations:** ^1^Department of Psychology, University of Oslo, Oslo, Norway; ^2^Consumption Research Norway, Oslo Metropolitan University, Oslo, Norway; ^3^Instituto Universitário de Lisboa, Centro de Investigação e de Intervenção Social, Lisbon, Portugal; ^4^Mannheimer Zentrum für Europäische Sozialforschung, University of Mannheim, Mannheim, Germany; ^5^Department of Anthropology, University of California, Los Angeles, Los Angeles, CA, United States

**Keywords:** baby schema, cuteness, kama muta, being moved, communal sharing, empathic concern, elevation, core values

## Abstract

A configuration of infantile attributes including a large head, large eyes, with a small nose and mouth low on the head comprise the visual baby schema or Kindchenschema that English speakers call “cute.” In contrast to the stimulus gestalt that evokes it, the evoked emotional response to cuteness has been little studied, perhaps because the emotion has no specific name in English, Norwegian, or German. We hypothesize that cuteness typically evokes kama muta, a social-relational emotion that in other contexts is often labeled in English as being moved or touched, heartwarming, nostalgia, patriotic feeling, being touched by the Spirit, the feels, etcetera. What evokes kama muta is sudden intensification of a communal sharing (CS) relationship, either CS between the person and another, or CS between observed others. In accord with kama muta theory, we hypothesize that a kama muta response to cuteness results from a sudden feeling of CS with the cute target. In colloquial terms, the perceiver adores the cute kittens and their heart goes out to them. When a person perceives cute targets interacting affectionately – that is, intensifying CS between them – this should strengthen a kama muta response. We experimentally investigated these predictions in two studies (*N* = 356). Study 1 revealed that videos of cute targets evoked significantly more kama muta than videos of targets that were not particularly cute. Study 2, pre-registered, found that, as hypothesized, when cute targets interacted affectionately they evoked more kama muta and were humanized more than when they were not interacting. We measured the level of kama muta by self-reports of sensations and signs and of feelings labeled *heartwarming, being moved*, and *being touched*. Participants’ ratings of kama muta were positively correlated with reported cuteness. In addition, as in our previous research on kama muta elicited by other types of stimuli, trait empathic concern predicted kama muta responses and perceived cuteness. The studies thus provide first evidence that cute stimuli evoke the heartwarming emotion of kama muta.

## Introduction

Cuteness overload: An overload of cuteness; when something or someone is so super cute that there is no word for it.

Urban Dictionary, 2008

Cute attack: A sensational response incited by the witnessing of something cute, precious, fuzzy, or otherwise snuggly. Symptoms include chills traveling up the spine and through the fingertips, impulsive smiling and jerking of the limbs. Severe cases of cute attacks can cause high-pitched squeals and temporary spasms of the entire nervous system, forcing its victim to crumble helplessly to the ground.

Urban Dictionary, 2009

Seeing something cute tends to evoke an emotion – an emotion with no name in English, German, or Norwegian, although others, such as the Uralic languages, do name it: *elérzékenyült* in Hungarian, *heldinud* in Estonian, *heltyä* in Finnish^1^. An emotional response to cuteness is widely recognized (if not named) by marketing professionals and utilized in commercial and charity advertising (Duffy and Burton, 2000; Nittono et al., 2012; Buckley, 2016; Nittono, 2016), environmental campaigns (Huddy and Gunnthorsdottir, 2000; Ruanguttamanun, 2014), and product design (Nenkov and Scott, 2014). Additionally, the Internet is filled with user-generated content of cute babies and animals that are evidently posted, viewed, shared, and liked because they evoke this emotion.^2^ There are people whose job it is to identify cute web content (Baron, 2014; Labato and Meese, 2014). Moreover, a positive affective response to cuteness is apparent in responses to the International Affective Picture System (IAPS), widely used in emotion research (Lang et al., 1997); the seven images rated highest in positive valence are all images of cute animals or human babies.

Cuteness is said to be one of the most fundamental influences on human behavior (Kringelbach et al., 2016; see also Dale, 2016). Although labels for it have been offered, such as “cuteness response” (Sherman and Haidt, 2011), “cute-affect,” “aww,” or “cute-emotion” (Buckley, 2016), the emotion that cuteness evokes has yet to be well-conceptualized or experimentally characterized. The current research aims to test the hypothesis that kama muta (Sanskrit for “moved by love”; Fiske et al., 2017a,c) is a particular emotion that people commonly experience in response to cute animals.

### What Cuteness Is, and What Emotion It Evokes

Lorenz (1943) described a configuration of infantile physical characteristics that he termed *Kindchenschema*, ‘baby schema’ (see also Glocker et al., 2009).^3^ A long line of psychological studies shows that when English speakers perceive beings that display such characteristics they label them *cute* (for example Pittenger, 1990; Gross, 1997; Volk et al., 2007). Stimuli such as human and animal infants draw attention, and people look at them longer than at less cute beings (Hildebrandt and Fitzgerald, 1978, 1981; Bellfield et al., 2011; Little, 2012; Golle et al., 2013; Borgi et al., 2014). Even 3 year-old children look longer at pictures of children with infantile features (Borgi et al., 2014).

Attentiveness to this configuration presumably is adaptive because it motivates responsiveness to the needs of one’s own offspring, and, in a few species, other infant close kin (Lorenz, 1943; Bradshaw and Paul, 2010; Leitão and Castelo-Branco, 2010; Sherman and Haidt, 2011). The needs of human infants are many and they depend on adults to fulfill these needs for an extraordinary long time. Thus, caretaking behavior can take many forms: for example hugging, feeding, playing, teaching, protecting, speaking, singing, looking, or smiling. The Kindchenschema configuration thus motivates caretaking in a broad sense, which has been repeatedly found (Volk et al., 2007; Glocker et al., 2009; Nittono et al., 2012; Sherman et al., 2013). For example, Volk et al. (2007) found that cuteness predicts willingness to adopt infants, while both Nittono et al. (2012) and Sherman et al. (2013) demonstrated that cuteness can increase carefulness (a proxy for caregiving behavior).

Historical changes in the design of children’s toys and cartoon characters reflect the attractiveness of the *Kindchenschema*. Over a period of 80 years, the design of Disney’s Mickey Mouse and the traditional stuffed teddy bear have each developed to fit Kindchenschema (Gould, 1980; Morris et al., 1995). Children between 6 and 8 years prefer teddy bears with such traits and display more caregiving behavior toward stuffed animals designed accordingly (Morris et al., 1995). Adults also prefer the Kindchenschema in human babies (Sanefuji et al., 2007). Nittono (2016) introduced a conceptualization of cuteness and a response to it with reference to the Japanese word ‘kawaii.’ In Japan, *kawaii* is culturally salient, highly elaborated, and highly motivating; women, in particular, generally aim to appear and act *kawaii*, and display many *kawaii* accouterments and household items. Nittono argues that the emotion evoked by kawaii is distinguished by moderate arousal, strong approach motivation, and “social orientation.”

However, to our knowledge, the only previous experimental research on the specifically emotional responses to cuteness is a set of studies by Aragón et al. (2015) and Aragón and Bargh (2018), who found that people display “dimorphous” emotional expressions to cute stimuli. That is, they found that cute stimuli evoked both care tendencies and behaviors that look like aggression, such as wishing to pinch, squeeze or bite the target, and clenching of hands and teeth.

The effect of cuteness on caretaking may be mediated by a certain kind of empathy, as this trait is thought to dispose one to altruistic behaviors such as caretaking (Batson et al., 2005). Batson et al. (2005) asked undergraduate participants to either read about a vulnerable protagonist (child, dog, or puppy) recovering from a broken leg, or read about a less vulnerable and less cute recovering adult. Cute vulnerable targets evoked stronger self-ratings of being *sympathetic, compassionate, tender, softhearted, warm*, and *moved*. These adjectives are thought to reflect the *empathic concern* state that is typically evoked by *responses to others in need* (Batson et al., 1987). This state has been hypothesized to reflect a parental caretaking response to vulnerable human babies (Niezink et al., 2012). Concordant with this hypothesis, Lishner et al. (2008) found that participants felt more empathic concern for human Kindchenschema faces and voices compared to adult counterparts. Similarly, Levin et al. (2017) demonstrated that reports of abuse of a child, puppy, or adult dog evoked more empathic concern and distress than reports of the same suffering of an adult human. Zickfeld et al. (2017a) found the same Kindchenschema effect on empathic concern for animal faces.

In sum, cute animals have facial features of the *Kindchenschema*, an evolved elicitor of attention, liking, approach, compassion, motivation to care for and protect one’s own infants and those of close kin. Humans thus seem to respond to cute animals in a similar way as to human infants, presumably triggered by the Kindchenschema. In addition, cute animals are perceived as vulnerable and needy, and people high in trait empathic concern seem to respond more strongly to cuteness than people low in empathic concern. Less consensus has been reached, however, about the emotional state evoked by perceiving cuteness. According to one theory, cuteness may evoke a specific positive emotion, kawaii, which motivates approaching others, while another approach suggests that cuteness tends to evoke a dimorphous response, which motivates both care and behaviors that look like aggression. We propose that cuteness evokes a specific positive emotion, kama muta, which motivates devotion to communal relations.

### Kama Muta

Kama muta theory postulates that a specific emotion, kama muta – which English speakers may label *feeling moved* or *touched* – occurs when a communal sharing (CS) relationship suddenly intensifies (Fiske et al., 2017a,c; see Zickfeld et al., in press for a review of research based on the vernacular lexeme, *moved*). Kama muta is a positive emotion that people actively seek out, like to evoke in other people, and want to experience together with others. Like other emotions, it varies in intensity.

The kama muta construct is based on relational models theory (RMT; Fiske, 1991, 1992, 2004). RMT postulates that people use four fundamental, biologically innate models to understand, motivate, evaluate, and coordinate nearly all social relationships and social structures. These four models are communal sharing, authority ranking, equality matching, and market pricing. Communal sharing refers to a group or dyadic social relationship in which participants have a sense of equivalence; their interaction is characterized by trust, unity, closeness, and kindness. Examples of CS include, but are not limited to, relationships between romantic partners and among family members. One can also form a communal relationship with non-human beings and with fictional characters (Fiske, 1991; Haslam, 2017), such as a cute animal, a teddy bear, or Mickey Mouse.

Kama muta theory (Fiske et al., 2017b,c) posits that the emotion which English speakers may label *being moved, touched, heartwarming, tenderness, nostalgia, team pride, patriotism, rapture, being touched by the Spirit, the feels, feeling stirred*, and other terms occurs when a CS relationship suddenly intensifies. This conceptualization has been confirmed by robust cross-correlational findings using the appraisal of increased social *closeness* as a measure of CS (Schubert et al., 2016). In addition, a study with 3542 paricipants in 19 nations responding in 15 languages using the KAMMUS scale to measure the appraisal of *suddenly increased communal sharing* along with other indicators of kama muta confirmed the substantial cross-correlation between these various indicators (Zickfeld et al., 2019). For example, the correlation of the *appraisal* scale with the *label* scale (self-report of being *moved, touched*, and *heartwarming*) was *r* = 0.54 [95% CI: 0.49, 0.59]. Additionally, the same study by Zickfeld et al. (2019) also provided discriminant validity of the KAMMUS scale as a measure of kama muta, distinct from amusement, sadness, and awe.

An increase in CS can be recognized subjectively as an increase in trust and feelings of unity with an interaction partner or a relationship partner, or it can be observed. Cues indicating increased CS include commensalism (eating together, feeding the other), touch, bodily proximity, synchrony and need-based giving (Fiske, 2004; Schubert et al., 2008). The suddenness of the appraisal can occur either as a sharp temporal transition from no relational model or another relational model to CS, or it can be against a backdrop of lack or loss of CS.

Kama muta theory further posits, and several studies show, that the emotion is characterized by certain sensations and signs. Such experiences typically involve a warm or other feeling in the center of the chest, goosebumps or chills, moist eyes or tears, a lump in the throat, feeling buoyant, being exhilarated, and sometimes also putting a hand to the chest, and saying something like “awww” or corresponding vocalizations in other languages (Zickfeld et al., 2019). Being in a state of kama muta is theorized to motivate caring and compassion and to be a highly positive occurrence that people actively seek out and are eager to share with others with whom they have a CS relationship (Fiske et al., 2017a,c). Accordingly, it is characterized by research participants as a predominantly positive experience whose motivational outcomes include wanting to hug someone, to share the experience again and do so together with others (Zickfeld et al., 2019). The kama muta construct has been conceptually and empirically distinguished from other, broader emotional valences such as happiness and sadness (Schubert et al., 2016; Fiske et al., 2017a,b,c; Seibt et al., 2017a,b, 2018; Zickfeld et al., 2017b).

Cuteness can evoke feelings closely related to kama muta. When Batson et al. (2005) asked participants to read about a cute, vulnerable protagonist (child, dog, or puppy), compared to narratives about less vulnerable and less cute targets, these targets evoked stronger ratings of *empathic concern* measured by self-reports of being sympathetic, compassionate, tender, softhearted, warm, and moved. Given the similar conceptualizations and operationalizations of empathic concern and kama muta, Zickfeld et al. (2017b) recently proposed that empathic concern is a trait that predicts how often and how intensely a person experiences kama muta, not only with regard to those who are in need, but across the whole spectrum of CS-intensifying events. Accordingly, their meta-analysis of 16 studies with United States and Norwegian participants found that the intensity of kama muta responses to video stimuli, as measured by ratings of *being moved or touched*, correlated 0.35 [95% CI: 0.29, 0.41] with trait empathic concern. In a subsequent 19-national study the overall correlation was 0.32 [95% CI: 0.28, 0.37] (Zickfeld et al., 2019). Both studies show that trait empathic concern is consistently related to three sensations and signs that are, together, a reliable indication of kama muta: feelings of warmth in the chest, positive tears, and goosebumps or chills.

What is the intensification of CS when a person reads or hears about, sees or interacts with a cute, vulnerable animal? We propose that perceiving cute animals activates the CS-model: a person feels affection, unity, closeness, and kindness toward that animal. Given that humans mainly relate in a CS way with other humans, we hypothesize that experiencing increased CS and kama muta in response to cute animals goes along with humanizing them. Kama muta thus is evoked by increased CS and reinforces devotion to that same CS relationship, for instance through caring for and protecting the animal, feeding and touching it, and being attentive to its expression of needs. We characterized this constellation of feeling kama muta about one’s own CS intensification as first person kama muta (Seibt et al., 2017a), i.e., as one’s ‘heart going out’ to the cute animal.

Conversely, third person kama muta is evoked by observing, reading or hearing about the CS intensification of others – such as videos of people showing exceptional love, kindness, or care for each other. We found that the more a person feels kama muta from watching third person CS intensifications, the more she tends to humanize the protagonists (Blomster et al., unpublished), and be motivated to engage in a CS relation with these protagonists (Zickfeld, 2015; Blomster et al., unpublished). Some of the videos that have been used to test kama muta theory involve animals showing care for each other (elephants) or for humans (a lion and a dog), and a human showing care for an animal (cat rescue) (Schubert et al., 2016; Seibt et al., 2017a,b).

Accordingly, we expect stimuli depicting individual cute animals to evoke first person kama muta (Study 1), and stimuli depicting animals interacting in a loving way to also evoke third person kama muta (Study 2). In this case, the interacting animals should also be perceived as cuter than non-interacting animals specifically because they evoke more kama muta. Our theory also predicts that the change in CS should be experienced as sudden in order to evoke kama muta. Therefore, our appraisal items (Zickfeld et al., 2019) tap into *sudden* change.

To summarize, kama muta theory posits that kama muta is an emotional response to an event in which a CS relationship suddenly intensifies. This emotion likely developed from parental and kin responses to small infants, facilitating care, compassion and protection, including hugging, feeding, defending the child and being responsive to its signals. Parental responses to small infants are triggered by the Kinchenschema, which humans perceive as cute. We therefore posit that animals high in cuteness should evoke the emotion of kama muta. Specifically, the central appraisal theme of kama muta, *suddenly increased CS*, is evoked either by the person’s ‘heart going out to the cute animal’ (first person) or by appraising the loving care that cute animals and their interaction partners display for each other (third person). The cues to increased CS relevant for the third person case that we manipulated in Study 2 are bodily proximity (cuddling, snuggling up, licking, touching) and feeding. These cues are universal signs of CS (Fiske, 2004).

### Overview of the Current Studies

We conducted two experiments to test whether cute features in animals (Study 1) and CS interactions among animals (Study 2) evoke the characteristic components of kama muta, including the typical labels, sensations and signs, motivations, and positive valence. To measure these components, we used a scale highly similar to the validated KAMMUS scale (Zickfeld et al., 2019). In a within-subject design in Study 1, participants were presented with videos of cute animals and animals that were not cute. We expected the cute animals to evoke more kama muta than the non-cute animals.

Kama muta is evoked by sudden intensification of CS, and is indexed by affectionate touching and feeding (Fiske, 2004). Hence, viewing cute targets’ affectionate touching and feeding should evoke stronger kama muta than the Kindchenschema alone. To test this proposition, Study 2 employed video stimuli of cute animals either interacting with each other in these CS ways or not interacting (but otherwise doing similar things) to manipulate increased CS between the target animals. We used the appraisal subscale of the KAMMUS to test whether the videos of the interacting animals are indeed appraised as a suddenly increased CS, and whether these appraisals correlate with the other components of kama muta. We expected that affectionate touching and feeding interaction between the targets would evoke both stronger ratings of cuteness and stronger kama muta emotion. We expected kama muta emotion to mediate the effect of CS content on cuteness perceptions. In Study 2, we also tested whether communally interacting cute animals are humanized more than non-interacting animals.

In both studies we tested whether trait empathic concern predicts kama muta responses to cuteness, just as it predicts kama muta responses to the other sudden intensifications of CS we have employed as stimuli.

The studies were approved by the internal review board of the Department of Psychology, University of Oslo. As recommended by Simmons et al. (2011), we report how we determined our sample size, all data exclusions, all manipulations, and all measures. All data sets, stimulus material and procedures are available at our OSF project page^4^.

## Study 1

The main objective of the first study was to experimentally investigate whether cute animals evoke the kama muta emotion more than animals that are minimally cute. The study tested the following two main hypotheses:^5^

**H1**: Viewing videos of cute animals, compared to videos of minimally cute animals, will evoke stronger kama muta ratings across four components of the emotion: vernacular labels, motivation to form or strengthen CS-relationships, emotional valence, and sensations and signs.

**H2**: Participants higher on trait empathic concern will rate the animals as cuter and will have higher ratings of kama muta in the four components.

### Methods

#### Participants

We recruited *N* = 121 participants through Amazon Mechanical Turk, requesting workers from the United States, and *N* = 176 Norwegian participants through convenience sampling on Facebook.^6^ Participants were excluded from the primary analyses based on the following *a priori* criteria; having more than 20% missing responses, not watching the videos, and being under the age of 18. Of the remaining *N* = 217^7^, *N* = 121 indicated that they were female (*N* = 3 indicated “other” or skipped that question), *N* = 105 were US American, *N* = 101 Norwegian, *N* = 11 from other countries or missing. Age varied from 19 to 63, *M* = 31.80, *SD* = 10.73; two participants did not provide demographic information.

#### Procedure and Materials

A within- and between-participants design was employed. Condition was a within-participants factor; participants saw both a video of a cute animal and a video of a non-cute animal. The order in which these videos were presented was randomized between participants. After each video, the participants were asked to rate the cuteness of the video and asked about their kama muta labels, valence, communal sharing motivation, and sensations and signs. Lastly, participants responded to the trait empathic concern measure, and provided demographic information.

The video stimuli comprised of eight pretested 20- to 40-s video clips depicting either very cute (e.g., bunny, kitten) or minimally cute animals (e.g., anglerfish, octopus, proboscis monkey). In each condition, participants saw one video randomly selected from a pool of four videos (see Supplementary Materials for video links and pre-test results).

#### Measures

The first and last author wrote a cuteness scale of nine items (e.g., “It is adorable”) to measure perceived cuteness of the animals in the videos. The scale was constructed based on a review of the literature, while attempting to identify the most distinctive and prevalent vernacular lexemes colloquially used to denote visual Kindchenschema cuteness. The cuteness scale included distractor items (not included in the number of items), and responses were assessed on a seven-point Likert scale from 0 (*not at all*) to 6 (*a lot*).

The experience of kama muta was assessed through four subscales, specifically: vernacular labels (six items: e.g., “I was moved”); sensations and signs (12 items: e.g., “A warm feeling in the center of the chest”); motivation to form or strengthen CS-relationships (seven items: e.g., “I felt more strongly committed to a relationship”); and emotional valence (two items: “I had positive feelings,” and “I had negative feelings”). The kama muta scale included distractor items (not included in the number of items), and offered response alternatives on a seven-point Likert scale from 0 (*not at all*) to 6 (*a lot*). This measure was an earlier version of the kama muta scale (*KAMMUS*) later validated in Zickfeld et al. (2019).

Empathic concern was measured with a subscale of the interpersonal reactivity index (IRI, Davis, 1980, 1983). Participants were asked to rate seven items such as “I am often quite touched by things that I see happen” on a five-point Likert scale, ranging from 1 (*does not describe me well*) to 5 (*describes me very well*). Item-level descriptive statistics and Norwegian translations for all measures can be found in the Supplementary Materials.

Lastly, participants were asked to indicate whether they listened to the sound of the video (which they had been instructed to turn off; see Supplementary Section “Study 1 Pretest of Video Stimuli” for further information) and provide demographic information, outlined in the participants section above.

### Results

We created five average scores from (1) six cuteness scale items (control condition: α = 0.91; cute condition: α = 0.94),^8^ (2) three kama muta vernacular labels items (control: α = 0.90; cute: α = 0.90), (3) 12 items of sensations and signs (control: α = 0.84; cute: α = 0.87), (4) four items of motivation (control: α = 0.95; cute: α = 0.94), and (5) the seven items of empathic concern (α = 0.88). The kama muta scores were constructed based on a subset of items validated in Zickfeld et al. (2019)^9^. We combined all sensation and sign items into one score. Analyses employing the separate sensation and sign factors are presented in the Supplementary Material.

#### Intercorrelations

We first assessed the co-occurrence among the four kama muta components, and the association of these aspects of kama muta with cuteness and empathic concern. In order to do so, we calculated intercorrelations of the cuteness scale, empathic concern, and the four kama muta components (vernacular labels, sensations and signs, motivations, and positivity) for the cute and non-cute conditions separately (see Table 1). Intercorrelations among the kama muta components were similar in the cute condition (*r*s between 0.48 and 0.79) and in the non-cute condition (*r*s between 0.45 and 0.77). The consistently strong correlations among the kama muta components support the validity of the kama muta construct, suggesting that these four components tap into the same construct.

**Table 1:** Study 1: intercorrelations of the kama muta components, cuteness ratings, and trait empathic concern in cute (left) and non-cute (right) conditions.

	Labels	Sensations and signs	Motivation	Positive valence	Cuteness
Sensations and signs	0.73^∗∗∗^/0.77^∗∗∗^				
Motivation	0.73^∗∗∗^/0.71^∗∗∗^	0.79^∗∗∗^/0.74^∗∗∗^			
Positive valence	0.48^∗∗∗^/0.47^∗∗∗^	0.57^∗∗∗^/0.45^∗∗∗^	0.48^∗∗∗^/0.48^∗∗∗^		
Cuteness	0.34^∗∗∗^/0.29^∗∗∗^	0.42^∗∗∗^/0.21^∗∗^	0.36^∗∗∗^/0.31^∗∗∗^	0.68^∗∗∗^/0.45^∗∗∗^	
Empathic concern	0.27^∗∗∗^/-0.01	0.28^∗∗∗^/0.04	0.31^∗∗∗^/0.08	0.43^∗∗∗^/0.04	0.43^∗∗∗^/-0.10


*^∗∗∗^*p* < 0.001, ^∗∗^*p* < 0.01 (two-tailed).*

In addition, all four kama muta components correlated strongly with perceived cuteness (*r*s between 0.34 and 0.68) in the cute condition. This correlational test of Hypothesis 1 supports the hypothesis that the emotion evoked by seeing cute animals is, in fact, kama muta. The four kama muta scores also correlated with empathic concern in the cute condition, supporting H2.

#### Main Analyses

We tested the hypotheses that cute animals would evoke more of all four components of kama muta than non-cute animals (H1), and that empathic concern would moderate the effect of condition (cute vs. non-cute) on kama muta ratings and cuteness ratings (H2). We did this by fitting mixed models using the *lme4* package in *R*.^10^ Both hypotheses were tested in five combined models, one for cuteness ratings and one for each of the four kama muta components. We regressed these dependent variables on the same set of predictors: condition, order of video, trait empathic concern, and all two-way interactions.^11^ For all models intercepts were allowed to vary randomly across participants and video. All factors were contrast coded and empathic concern was mean-centered. We report unstandardized effect size estimates *B* and their 95% confidence intervals. In addition, we report standardized effect sizes (Cohen’s *d* or Pearson’s *r*) for all main effects. Table 2 provides an overview of all models.

**Table 2:** Study 1: prediction of cuteness, moved, sensations and signs, motivation, and positive valence by condition, order, empathic concern (EC), and their two-way interactions using mixed models.

Predictor	*F*	df1,df2	*p*	B [95% CI]	*d* (*r*^†^)
	Cuteness (Model 1)				
Condition	122.43	1,6	<0.001	3.85 [3.18, 4.51]	3.04
Order	25.09	1,209	<0.001	-0.50 [-0.69, -0.30]	-0.22
EC	33.21	1,211	<0.001	0.37 [0.25, 0.50]	0.15^†^
Condition∗Order	5.94	1,211	0.02	0.58 [0.11, 1.04]	–
Condition∗EC	36.37	1,210	<0.001	0.66 [0.44, 0.87]	–
Order∗EC	0.53	1,209	0.468	0.08 [-0.13, 0.30]	–
	Labels (Model 2)				
Condition	104.84	1,4	<0.001	1.61 [1.31, 1.92]	1.15
Order	4.77	1,207	0.03	-0.23 [-0.43, -0.02]	-0.14
EC	12.89	1,212	<0.001	0.30 [0.14, 0.46]	0.17^†^
Condition∗Order	2.62	1,213	0.107	0.49 [-0.10, 1.09]	–
Condition∗EC	27.06	1,208	<0.001	0.60 [0.37, 0.82]	–
Order∗EC	0.01	1,207	0.922	-0.01 [-0.23, 0.22]	–
	Sensations and signs (Model 3)				
Condition	53.83	1,4	0.001	0.57 [0.42, 0.72]	0.74
Order	4.12	1,206	0.04	-0.10 [-0.20, -0.005]	-0.13
EC	9.65	1,212	0.002	0.15 [0.06, 0.25]	0.17^†^
Condition∗Order	3.59	1,212	0.06	0.34 [-0.01, 0.69]	–
Condition∗EC	20.98	1,207	<0.001	0.25 [0.14, 0.36]	–
Order∗EC	0.66	1,206	0.419	0.04 [-0.06, 0.15]	–
	Motivation (Model 4)				
Condition	28.00	1,4	0.005	0.79 [0.49, 1.09]	0.65
Order	0.01	1,207	0.929	-0.01 [-0.17, 0.15]	-0.01
EC	10.68	1,213	0.001	0.25 [0.10, 0.40]	0.18^†^
Condition∗Order	4.65	1,213	0.03	0.60 [0.06, 1.14]	
Condition∗EC	18.62	1,208	<0.001	0.39 [0.21, 0.56]	
Order∗EC	0.40	1,207	0.527	0.06 [-0.12, 0.24]	
	Positive valence (Model 5)				
Condition	61.06	1,5	<0.001	2.17 [1.63, 2.71]	1.18
Order	4.16	1,208	0.04	-0.27 [-0.53, -0.01]	-0.13
EC	22.62	1,213	<0.001	0.51 [0.30, 0.72]	0.22^†^
Condition∗Order	11.45	1,213	<0.001	1.32 [0.56, 2.08]	–
Condition∗EC	32.01	1,209	<0.001	0.83 [0.54, 1.11]	–
Order∗EC	0.12	1,208	0.729	0.05 [-0.23, 0.34]	–


*All outcome variables were measured on scales from 0 to 6. All factors were contrast coded (condition: -0.5 = control; 0.5 = cute, order: -0.5 = first; 0.5 = second). The covariate (EC) was measured on a scale from 1 to 5 and mean centered. For all models intercepts were allowed to vary randomly across participants and video. Values denoted with ^†^ represent correlation coefficients.*

First, we observed a main effect of condition for all five models, as seen in Table 2. Validating our experimental manipulation, we observed that in Model 1 the high cuteness videos induced higher cuteness ratings than low cuteness videos (see Table 3 for descriptive statistics). In addition, supporting our first hypothesis, we found that in Model 2, ratings of kama muta labels were higher for the high cuteness videos compared to the low cuteness videos. Similarly, participants reported more kama muta sensations and signs in Model 3 for the high cuteness videos than for the low cuteness videos. In Model 4, ratings for the CS motivation component of the kama muta emotion were also higher in the high cuteness videos in contrast to the non-cute videos. Finally, in Model 5, participants rated the high cuteness videos as more positive than the low cuteness videos.

**Table 3:** Descriptive statistics for the kama muta components, cuteness ratings, and trait empathic concern in Study 1.

	Cute		Non-cute	
	
Scale	M (SE)	95% CI	M (SE)	95% CI
Labels	2.07 (0.11)	[1.81, 2.34]	0.49 (0.11)	[0.23, 0.76]
Sensations and Signs	0.93 (0.06)	[0.79, 1.08]	0.38 (0.06)	[0.24, 0.53]
Motivation	1.15 (0.12)	[0.88, 1.43]	0.37 (0.12)	[0.10, 0.65]
Positive valence	4.07 (0.19)	[3.61, 4.52]	1.95 (0.19)	[1.49, 2.40]
Cuteness	4.72 (0.25)	[4.12, 5.32]	0.94 (0.25)	[0.34, 1.55]
Empathic concern	3.80 (0.04)	[3.72, 3.89]	3.80 (0.04)	[3.72, 3.89]


*Participants were asked to indicate their agreement on scales ranging from 0 to 6, with the exception of empathic concern which was rated from 1 to 5. Empathic concern was measured once, hence the same values in both conditions.*

Supporting our second hypothesis, trait empathic concern positively predicted ratings of cuteness, and ratings of all four components of kama muta. For all models we observed an interaction effect of empathic concern with condition: the effects of trait empathic concern were stronger for the high cuteness videos (see Table 1 for intercorrelations of empathic concern and cuteness). Finally, we found a main effect of order of video for all models except the sensations and signs model. For these three models, ratings were stronger for the first video. We also observed an interaction effect between order and condition in the cuteness, motivation, and positive valence model: the effect of video order on the low cuteness videos was strongest.^12^ We did not detect a significant interaction effect between empathic concern and order for any of the models.

### Discussion

Hypothesis 1 was supported. Participants’ ratings of all four kama muta components were higher when watching the cute videos, compared to the non-cute videos: cuteness evoked significantly stronger motivation to engage in CS-relationships; more intense sensations and signs; more subjective feelings of being *moved, touched*, and *heart-warmed;* and more positive feelings. These data support the theory that cuteness evokes kama muta in the perceiver.

Participants higher on trait empathic concern also had higher ratings on all of the kama muta components and on cuteness, supporting H2. In addition, we found an interaction effect of empathic concern and condition in all models, meaning that participants higher on EC were more sensitive to the cute videos, rating these as cuter and more kama muta evoking.

## Study 2

Study 1 established that images of cute animals evoke kama muta. Study 2 tested whether adding well-established signs of CS to these stimuli, namely affiliative contact between the cute animals or feeding them results in greater cuteness perception and stronger kama muta reactions, compared to videos of cute animals not interacting and not being fed. A second objective of this study was to investigate whether kama muta responses mediated the effect of the touching and feeding CS manipulations on cuteness perception. Hence, we preregistered the following six hypotheses (https://osf.io/bjuva/):^13^

**H1:** High communal sharing (CS) videos will be judged as cuter than low CS videos.

**H2:** Compared to low CS videos, high CS videos will evoke more kama muta, as measured by (a) the kama muta sensations and signs, and (b) labels.

**H3**: The effect of CS on cuteness ratings will be mediated by kama muta, as measured by (a) the kama muta labels and (b) sensations and signs.

**H4:** Trait empathic concern positively predicts cuteness ratings.

**H5:** High CS videos will lead to more perceived humanness of the animal protagonists than the low CS videos.

**H6:** Humanness ratings of the animals and kama muta evoked will correlate.

### Methods

#### Participants

We conducted an *a priori* power analysis based on an effect size of *f* = 0.15 (α = 0.05, 1-β = 0.95), which suggested a total sample size of 148 participants^14^. We recruited *N* = 201 participants in Norway through convenience sampling on Facebook and a student research participation pool at the University of Oslo where students were invited to participate in a study investigating emotional responses to video stimuli. As pre-registered, participants were excluded from the primary analyses if they indicated participating for their personal educational purposes only (i.e., choosing not to contribute their data to the study), having more than 20% missing values, and not watching the whole video. Of the remaining *N* = 139,^15^ *N* = 107 indicated that they were female (*N* = 1 indicated “other”), *N* = 130 were Norwegian, *N* = 9 from other countries. Age varied from 16 to 63, *M* = 24.28, *SD* = 7.58.

#### Materials and Procedure

A mixed design was employed. Condition was a within-subjects factor where participants saw a video of two subjects (either two animals or one animal and one human^16^) engaging in an affectionate interaction like cuddling, liking, and feeding one another (high CS), and a video of two subjects interacting minimally or not at all (low CS). Another within-subjects factor was type of animal: participants saw one video featuring dogs and one video featuring cats. The between-subjects factors were the video version (Video A or B of a particular stimulus set), and the order in which the videos were presented (high CS first or high CS second).

We created four stimulus sets (high or low CS video with cats or dogs) with two videos each (see Supplementary Materials for links to the eight videos). The videos were pairwise matched between CS conditions, meaning that apart from the CS manipulation, everything else was held constant (i.e., the targets, the movement of the targets, background, setting, and lighting). Two videos were sampled per participant. The first video was sampled from one of the four stimulus sets and the second video was then sampled from the stimulus set of the other CS condition and other animal.

After each video, participants were asked to rate the cuteness of the video, the perceived humanness of the animal subject(s), and five aspects of their own kama muta emotion. Finally, participants responded to the trait empathic concern items, and provided demographic information.

#### Measures

A revised six-item scale from Study 1 measured perception of cuteness; 3 negatively worded items (e.g., “The video was not cute”) and 3 positively worded items (e.g., “The video was adorable”). The experience of kama muta was assessed through five subscales using an earlier version of the KAMMUS that has since been further validated in Zickfeld et al. (2019), specifically: vernacular labels (7 items, same as in Study 1 with the addition of “I felt in love”); sensations and signs (14 items, with “choked up” and “difficulty speaking” added as additional items from the subscale in Study 1); CS intensification appraisals (10 items: e.g., “I observed a special sense of belonging”); motivation to form or strengthen CS-relationships (7 items, same as in Study 1 but one item rephrased to “I felt especially friendly” from “I felt especially friendly to nearly everyone); and emotional valence (two items: “I had positive feelings,” and “I had negative feelings”). The cuteness and kama muta scales included distractor items (not included in the number of items), which were, as planned, excluded from all analyses. A single item written by the third author was added to the cuteness scale to assess humanization of the animal protagonist(s) in the videos: “The animal(s) in the video seemed human to me.” Answers were given on a seven-point Likert scale from 0 (*not at all*) to 6 (*a lot*). The same empathic concern measure and demographic questions used in Study 1 were again presented in Study 2.

### Results

We created six average scores (1) from the six cuteness scale items (low CS condition: α = 0.75; high CS condition: α = 0.59); (2) from the three kama muta vernacular labels (low CS: α = 0.85; high CS: α = 0.85); (3) the 12 items of sensations and signs (low CS: α = 0.79; high CS: α = 0.89); (4) the four items of CS intensification appraisals (low CS α = 0.95; high CS α = 0.94); (5) four items of motivation (low CS: α = 0.92; high CS: α = 0.91), and (6) the seven items of empathic concern (α = 0.76). As in Study 1, the KAMMUS subscales were constructed based on Zickfeld et al. (2019) but with only one score for all sensations and signs combined; analyses employing the separate sensation and sign factors are presented in the Supplementary Material.

#### Intercorrelations

Correlations among the main variables are presented in Table 4. Ratings of perceived humanness in the animals correlated positively with all kama muta components (*r*s between 0.28 and 0.52), supporting H6. As in Study 1, all of the kama muta factors correlated with all of the others in both the low and high CS condition (*r*s between 0.42 and 0.79). We also observed positive correlations between the cuteness scale and all five kama muta indicators. Finally, as in Study 1, empathic concern correlated more with all other variables in the experimental (i.e., high CS) condition, compared to the low CS condition.

**Table 4:** Study 2: intercorrelations of the kama muta components, cuteness ratings, trait empathic concern, and humanness in the high CS (left) and low CS (right) conditions.

	Communal sharing	Labels	Sensations and signs	Motivation	Positive valence	Cuteness	Empathic concern
Labels	0.63^∗∗∗^/0.79^∗∗∗^						
Sensations and signs	0.53^∗∗∗^/0.59^∗∗∗^	0.72^∗∗∗^/0.73^∗∗∗^					
Motivation	0.56^∗∗∗^/0.61^∗∗∗^	0.59^∗∗∗^/0.66^∗∗∗^	0.58^∗∗∗^/0.58^∗∗∗^				
Positive valence	0.57^∗∗∗^/0.49^∗∗∗^	0.57^∗∗∗^/0.68^∗∗∗^	0.42^∗∗∗^/0.51^∗∗∗^	0.46^∗∗∗^/0.45^∗∗∗^			
Cuteness	0.47^∗∗∗^/0.35^∗∗∗^	0.53^∗∗∗^/0.46^∗∗∗^	0.41^∗∗∗^/0.39^∗∗∗^	0.36^∗∗∗^/0.38^∗∗∗^	0.56^∗∗∗^/0.56^∗∗∗^		
Empathic concern	0.15^∗^/0.00	0.24^∗∗^/-0.01	0.24^∗∗^/0.06	0.18^∗^/0.04	0.22^∗∗^/0.02	0.42^∗∗∗^/0.11	
Humanness	0.52^∗∗∗^/0.50^∗∗∗^	0.44^∗∗∗^/0.44^∗∗∗^	0.47^∗∗∗^/0.33^∗∗∗^	0.32^∗∗∗^/0.33^∗∗∗^	0.28^∗∗∗^/0.31^∗∗∗^	0.17^∗∗^/0.20^∗^	0.15^∗^/0.14


*^∗^*p* < 0.05, ^∗∗^*p* < 0.01, ^∗∗∗^*p* < 0.001 (two-tailed). Also note that sample sizes for the correlations differ slightly because of missing values.*

#### Main Effect Analyses

We used a series of mixed models to test the hypotheses.^17^ The final dataset consisted of a total of 278 video reactions. For all models, intercepts were allowed to vary randomly across participants. We regressed each dependent variable (CS-intensification appraisals, cuteness, humanness, kama muta labels, sensations and signs, motivation, and positivity) in a separate model on the same set of predictors: cuteness condition, type of animal presented, order of video, and video version, as well as interactions between condition and order, and between animal type and version. For the cuteness model, we added trait empathic concern as a covariate. All factors were contrast coded (see Table 5) and empathic concern was mean-centered. We report unstandardized effect size estimates *B* and their 95% confidence intervals. In addition, we report standardized effect sizes (Cohen’s *d* or Pearson’s *r*) for all main effects. Table 5 gives an overview of all models.

**Table 5:** Study 2: prediction of the individual kama muta components, cuteness, and humanness by animal type, order, video version, empathic concern (EC) and the Interactions Condition × Order, and Animal Type × Version Using Mixed Models.

Predictor	*F*	df1,df2	*p*	B [95% CI]	*d* (*r*†)
	Communal sharing (Model 1)				
Condition	107.54	1,134	<0.001	1.58 [1.28, 1.88]	1.02
Animal type	0.46	1,133	0.499	0.10 [-0.19, 0.40]	0.06
Order	3.17	1,133	0.08	-0.27 [-0.57, 0.02]	-0.16
Video version	0.13	1,254	0.715	0.06 [-0.28, 0.41]	0.04
Condition∗Order	0.69	1,137	0.406	-0.35 [-1.17, 0.47]	–
Animal Type∗Version	9.69	1,232	0.002	1.07 [0.40, 1.74]	–
	Cuteness (Model 2)				
Condition	50.50	1,133	<0.001	0.79 [0.57, 1.00]	0.73
Animal type	4.43	1,133	0.037	-0.23 [-0.45, -0.02]	-0.20
Order	8.51	1,133	0.004	-0.32 [-0.54, -0.11]	-0.29
Video version	0.75	1,260	0.388	0.11 [-0.13, 0.34]	0.09
EC	13.19	1,133	<0.001	0.39 [0.18, 0.59]	0.22†
Condition∗Order	0.007	1,135	0.931	0.02 [-0.51, 0.56]	–
Animal Type∗Version	0.30	1,244	0.584	-0.13 [-0.60, 0.34]	–
	Labels (Model 3)				
Condition	26.95	1,133	<0.001	0.70 [0.44, 0.96]	0.44
Animal type	0.43	1,133	0.515	-0.09 [-0.35, 0.17]	-0.05
Order	6.71	1,133	0.01	-0.35 [-0.61, -0.09]	-0.22
Video version	1.03	1,227	0.310	-0.17 [-0.50, 0.16]	-0.11
Condition∗Order	0.52	1,136	0.472	-0.33 [-1.23, 0.57]	–
Animal Type∗Version	1.33	1,206	0.249	0.37 [-0.26, 1.01]	–
	Sensations and signs (Model 4)				
Condition	9.88	1,124	0.002	0.26 [0.10, 0.42]	0.30
Animal type	0.04	1,124	0.842	-0.02 [-0.18, 0.14]	-0.02
Order	3.67	1,124	0.06	-0.16 [-0.32, 0.002]	-0.18
Video version	0.35	1,231	0.552	-0.06 [-0.25, 0.14]	-0.07
Condition∗Order	0.27	1,129	0.602	-0.13 [-0.62, 0.36]	–
Animal Type∗Version	0.46	1,208	0.497	0.13 [-0.24, 0.51]	–
	Humanness (Model 5)				
Condition	4.62	1,134	0.03	0.32 [0.03, 0.62]	0.18
Animal type	2.50	1,134	0.12	0.24 [-0.05, 0.53]	0.13
Order	3.81	1,134	0.053	-0.29 [-0.59, -0.002]	-0.16
Video version	0.20	1,223	0.66	0.08 [-0.28, 0.45]	0.05
Condition∗Order	1.70	1,137	0.20	-0.68 [-1.71, 0.35]	–
Animal Type∗Version	1.01	1,204	0.32	0.36 [-0.34, 1.07]	–
	Motivation (Model 6)				
Condition	15.87	1,133	<0.001	0.52 [0.27, 0.77]	0.32
Animal type	5.06	1,133	0.03	-0.29 [-0.55, -0.04]	-0.18
Order	9.76	1,133	0.002	-0.41 [-0.66, -0.16]	-0.25
Video version	0.05	1,217	0.818	-0.04 [-0.36, 0.29]	-0.02
Condition∗Order	0.14	1,136	0.713	-0.18 [-1.11, 0.76]	–
Animal Type∗Version	2.59	1,197	0.109	0.51 [-0.11, 1.13]	–
	Positive valence (Model 7)				
Condition	36.98	1,131	<0.001	1.02 [0.70, 1.35]	0.60
Animal type	6.01	1,131	0.02	-0.41 [-0.74, -0.09]	-0.23
Order	4.51	1,131	0.04	-0.36 [-0.69, -0.03]	-0.20
Video version	1.25	1,255	0.265	-0.22 [-0.60, 0.16]	-0.12
Condition∗Order	0.26	1,134	0.611	-0.24 [-1.15, 0.67]	–
Animal Type∗Version	0.00	1,232	0.990	-0.005 [-0.77, 0.76]	–


*All outcome variables were measured on scales from 0 to 6. All factors were contrast coded (condition: -0.5 = low CS, 0.5 = high CS; animal type: -0.5 = cat, 0.5 = dog; order: -0.5 = first, 0.5 = second; video version: -0.5 = version 1, 0.5 = version 2). The covariate (EC) was measured on a scale from 1 to 5 and mean centered. For all models intercepts were allowed to vary randomly across participants. Values denoted with † are correlation coefficients.*

Seen in Model 1 of Table 5, the main effect of condition on CS-intensification ratings was significant; high-CS videos were rated higher on CS intensification appraisals than low CS videos. The manipulation was therefore successful (for descriptive statistics see Table 6). In addition, we observed an interaction effect between type of animal and video version on the CS appraisals. The second version of the cat video evoked less CS appraisals than all other videos.

**Table 6:** Descriptive statistics for the kama muta components, cuteness ratings, humanness, and trait empathic concern in Study 2.

	High CS		Low CS	
		
Scale	M (SE)	95% CI	M (SE)	95% CI
Communal sharing	2.90 (0.13)	[2.65, 3.16]	1.30 (0.13)	[1.04, 1.56]
Labels	2.31 (0.13)	[2.05, 2.57]	1.60 (0.13)	[1.33, 1.86]
Sensations and signs	2.31 (0.13)	[2.05, 2.57]	1.60 (0.13)	[1.33, 1.86]
Motivation	1.92 (0.14)	[1.64, 2.19]	1.36 (0.14)	[1.09, 1.63]
Positive valence	4.40 (0.14)	[4.12, 4.69]	3.36 (0.14)	[3.08, 3.64]
Cuteness	4.90 (0.09)	[4.72, 5.08]	4.08 (0.09)	[3.90, 4.26]
Humanness	2.55 (0.15)	[2.25, 2.85]	2.21 (0.15)	[1.91, 2.51]
Empathic concern	3.96 (0.05)	[3.85, 4.07]	3.96 (0.05)	[3.85, 4.07]


*Participants were asked to indicate their agreement on scales ranging from 0 to 6, with the exception of empathic concern, which was from 1 to 5. Empathic concern was measured once, hence the same values in both conditions.*

Seen in Model 2, cuteness ratings were higher in the high-CS videos in contrast to the low-CS videos, supporting H1. Supporting H4, we also observed a positive effect of trait empathic concern on the cuteness ratings. ^18^ There was also a significant main effect of animal type (cats were rated as cuter than dogs).

Seen in Model 3, participants’ ratings on the kama muta labels were higher in the high CS condition than in the low CS condition (see Table 6). The same was true for the kama muta sensations and signs, as seen in Model 4. Both models support H2.

Seen in Model 5, participants rated the animals in the high CS condition as more human than animals in the low CS condition, therefore supporting H5.

Finally, we also explored whether condition influenced CS motivation and positive valence ratings. We observed that motivation ratings in Model 6 were higher in the high CS condition compared to the low CS condition. We also observed in Model 7 that ratings of positive valence were higher in the high CS condition than in the low CS condition.

In Models 2, 3, 6, and 7 we also found an order effect where the first video had higher ratings than the second video on each of the kama muta components and on cuteness. Order did not interact with condition in any of the models, thus, the order effects do not invalidate the conclusions from the hypothesis tests.

#### Mediation Analyses

**FIGURE 1:**
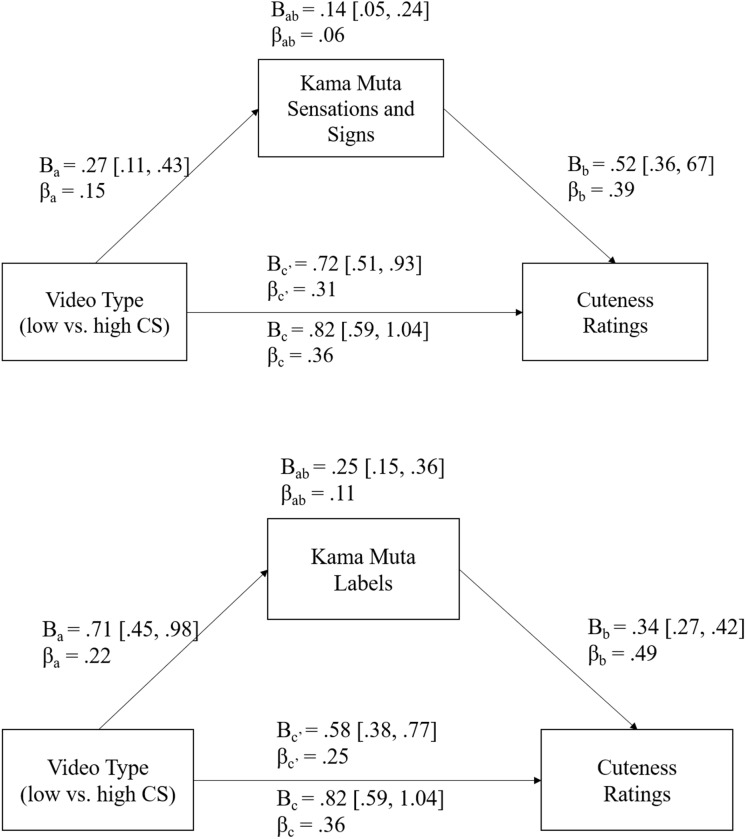
Mediation analyses of H3. Path diagram showing the direct (c′), indirect (a^∗^b), and total unstandardized (B) and standardized (β) effect (c) of video content on cuteness ratings and its partial mediation of the kama muta sensations and signs (model 1), and the kama muta labels (model 2).

Mediation analyses were conducted to test H3, that the effect of high or low CS (video condition) on cuteness ratings (as revealed by H1) was mediated by kama muta, as measured by the sensations and signs (Model 1) and labels (Model 2, see Figure 1).

The possible mediation by kama muta was tested using three mixed models (Bauer et al., 2006). To obtain path *a*, a mixed regression of the mediator on the independent variable was performed. Paths *b* and c′ were determined by regressing the dependent variable on the mediator and the independent variable. To obtain path *c*, we regressed the dependent variable on the independent variable. Coefficients for the different paths and the indirect effect were manually calculated and standardized according to Bowman (2012), while a confidence interval for the indirect effect was estimated using a Monte Carlo simulation method (Falk and Biesanz, 2016).^19^

As seen in Model 1 of Figure 1, kama muta sensations and signs mediated the relationship between low and high CS condition and cuteness ratings. Model 2 of Figure 1 showed that kama muta labels also mediated the relationship between CS condition and cuteness ratings. Both the sensations and signs and the labels partially mediated the main effect of condition on cuteness ratings; the direct effect of condition on the cuteness ratings remained strong. Thus, high CS videos (showing two animals affectionately interacting with each other or feeding) received higher kama muta ratings, which then increased participants’ perceptions of the cuteness of the animals. However, kama muta does not account for the whole effect of condition on cuteness.

### Discussion

Study 2 showed that when seeing two cute animals interacting affectionately, participants rated them as cuter and more human. They also evoked more kama muta, as indexed by the use of vernacular labels for kama muta, by reporting more sensations and signs typical of kama muta episodes, by indicating the experience as being more positive and by feeling motivated to connect in a CS way. As in Study 1, participants higher on trait empathic concern were more inclined to rate the animals in the videos as cute. Lastly, we found that the difference in cuteness ratings between the high and the low CS conditions was partly explained by increased kama muta in the high CS condition. Therefore, all hypotheses were supported in Study 2. However, we found order effects where the first video was consistently rated as cuter or evoking more kama muta than the second video. Given that this effect did not interact with condition, it does not compromise our conclusions.

## General Discussion

Two studies with a total of 356 participants supported the hypothesis that cuteness evokes *kama muta*, a social-relational emotion that, in other contexts, is often labeled in English *moved, touched, heartwarming, nostalgia, patriotism, team spirit, feeling God’s love*, etcetera. In both studies, we presented videos of animals differing in cuteness and observed stronger ratings of four aspects of kama muta in response to the cuter category. The four indicators or components we assessed were the use of kama muta labels to describe one’s emotional response, the judged positivity of that response, the motivation to connect to others in a communal way, and the report of typical sensations and signs of kama muta, such as warm feelings in the chest, tears, or goosebumps. Moreover, within each stimulus category, we observed significant correlations between the judged cuteness of each stimulus and the four components of the kama muta response. Furthermore, across both studies, we observed that the empathic concern trait predicted ratings of cuteness and kama muta responses to them, corroborating research from Lehmann et al. (2013) and Zickfeld et al. (2017b, 2019). This confirmed our hypothesis that empathic concern, as a general predisposition for feeling kama muta, would also predict kama muta responses to cuteness.

Since many studies have shown that kama muta is evoked by the observation of a sudden intensification of CS in others, we further hypothesized that kama muta responses to cuteness would be strongest when observing affectionate contact between the target animals or the target animal and a human hand. Study 2 confirmed this with respect to four aspects of kama muta. Given that persons perceive CS relations as an important part of human nature (Haslam, 2006), we also expected that participants would humanize the affectionately interacting animals more than the non-interacting animals, and that a stronger kama muta response would go along with more humanization of the animals. We also expected that the interacting animals would be judged as cuter than their non-interacting counterparts, and kama muta responses would mediate this effect of affectionate interaction on cuteness ratings. Results of Study 2 supported these hypotheses.

### Kama Muta Is a Typical Response to Cuteness

The first study demonstrated that compared to videos of less cute animals, videos of cute animals evoked significantly more intense sensations and signs of kama muta, a stronger motivation for communal interactions, more positive feelings, and higher ratings on labels relevant to kama muta (*moved, touched* and *heart-warming*). This finding complements that of Batson et al. (2005), who showed that cuteness (of a dog, puppy or child) evoked subjective feelings labeled being *moved*. Going beyond Batson and colleagues findings, the present findings indicate that one cuteness response is kama muta, by providing evidence for the various components typical of an emotional episode – not only a label but also sensations and signs, an appraisal, and a motivational tendency (Moors et al., 2013). Americans and Norwegians evidently feel kama muta in response to cuteness, despite the fact that in this context, they can’t readily name their emotion (Fiske et al., 2017c). Other languages do have a distinct, accessible, consensual name for kama muta in response to cuteness, or else use the same lexeme they use for kama muta in other contexts. Even though the cuteness scores were generelly high, the mean ratings for the sensations and signs, motivations, and labels in response to cuteness were all found to be relatively low. Nevertheless, we did find a significant difference in all kama muta components between the experimental and control conditions. This indicates that kama muta is a typical response to cuteness. Of course, it is likely to be stronger in direct interactions with living cute targets.

Consistent with all our previous research showing that trait empathic concern correlates with kama muta response states, Study 1 showed that empathic concern moderated the relationship between condition and rating of the different kama muta components, meaning that people high on trait EC reported feeling more kama muta in the cute condition than people lower on trait EC. Kama muta motivates compassion, care, and solidarity, including, we suggest, the motivation to respond to the needs of cute human and non-human infants.

Indeed, precisely because of this, we speculate that the phylogenetic source of kama muta is maternal bonding. Mothers must instantly form intense CS bonds to offspring at the moment of birth. In the small percent of species that form pair bonds and the smaller percentage in which siblings and other kin contribute to care of the infant, the father and those kin, too, must instantaneously form CS bonds with the infant. Thus we concur with McDougall who described the *tender emotion* (one of the seven basic emotions) – something very much like kama muta – as an outgrowth of the human maternal instinct to care for their own babies, extended to an emotion experienced in a vast array of eliciting situations:

In the human being, just as is the case in some degree with all the instinctive responses... there takes place a vast extension of the field of application of the maternal instinct. The similarity of various objects to the primary or natively given object, similarities which in many cases can only be operative for a highly developed mind, enables them to evoke tender emotion and its protective impulse directly.

McDougall, 1919, pp. 57–58, see also McDougall, 1923

#### Communal Sharing Mediates a Kama Muta Response to Cuteness

While Study 1 showed that cuteness evokes kama muta, apparently through first-person CS with the cute targets, the second study revealed that the kama muta response, along with cuteness ratings, were significantly larger when the participants observed CS intensification. That is, observing an affectionate interaction between two cute animals, or between a cute animal and a human hand, evoked third-person kama muta in addition to the first-person kama muta evoked by observing the same two protagonists when they were not interacting. This may explain why online video content of cute animals typically includes a caring interaction, often cuddling or caressing. Witnessing a caring and tender relationship between others is typically *moving* and *heart-warming* in itself, even when the protagonists are not cute (Schubert et al., 2016; Seibt et al., 2017a,b). Nittono and Ihara (2017) have shown that cute images typically elicit facial muscles associated with smiling. Smiling often occurs in communal feelings, especially when they intensify, and is a common (though not unique/distinctive) reaction to kama muta experiences (Zickfeld, 2015; Zickfeld et al., 2019). Earlier studies on the kama muta emotion have found that appraisals of sudden CS intensification are strong predictors of a kama muta experience (Zickfeld et al., 2019). In conjunction with the results presented here, this further validates kama muta as a cuteness emotion: when we increased the kama muta response to cute animals by showing them interacting communally, we combined two sources of kama muta responses (first and third person), which resulted in even stronger kama muta responses and ratings of the animals as cute. However, the partial mediation effect found in Study 2 suggests that other factors than CS may have additional influences on cuteness responses, such as preferences and attractions for different animals, and various personality traits.

#### Cuteness, Kama Muta, and Empathic Concern

Results from the present studies provide evidence that individuals scoring high on empathic concern, the tendency to express sympathy for others in need (Davis, 1983), report stronger experiences of kama muta and cuteness. While previous studies have consistently shown that cute features in animals or infants evoke strong experiences of *empathic concern* (Batson et al., 2005; Zickfeld et al., 2017a), there are reasons to believe that the feeling of empathic concern is a motivational facet of kama muta (Zickfeld et al., 2017b, 2019). This is not surprising as state empathic concern has been assessed using labels such as *moving* or *warmth*, which are the most common English labels for kama muta. Niezink et al. (2012) have provided evidence that empathic concern consists of aspects of *sympathy* and *tenderness*. While we have argued that the sympathy component might evoke kama muta through identification with the target in need (Zickfeld et al., 2017b), the present Study 2 suggests that intensifications in CS increase experiences of cuteness – the tenderness component of empathic concern. The present research provides further evidence that kama muta and empathic concern are highly intertwined and play a central role in cuteness experiences.

#### Kama Muta and Humanization

The results of Study 2 showed that cute animals interacting communally were seen as more human than cute animals not interacting, albeit with a small effect size (*d* = 0.18). In addition, the inter-correlations in Table 4 show that all kama muta components correlated moderately with humanness ratings within both conditions, and that these correlations were highest between appraisals of CS intensification and humanness. This gives further support to the notion that perceiving interactions as communal makes the agents seem more human. We believe that this occurs because acting communally shows that the agents are able to care for each other, which people construe as a core aspect of humanness (Opotow, 1990). Similarly, Blomster et al. (unpublished) found that out-group members interacting communally and therefore eliciting kama muta in participants (as compared to acting in a manner that elicits amusement) were perceived as more human. Moreover, the same study also found that humanness ratings of out-group members before the kama muta manipulation predicted how much kama muta participants felt, suggesting a bidirectional relationship between kama muta and humanization. Therefore, future studies should investigate whether people who perceive animals as less different from humans (see Hodson et al., 2015) are more susceptible to feeling kama muta toward cute animals. In other words, is there a bidirectional relationship between kama muta and humanness for cute animals?

### Limitations

The results of the current studies should be considered in light of their limitations. As reported the results section, order effects were detected in both studies. This might be due to anchoring effects. This fits the actual pattern of the means of cuteness, showing that when a low cuteness video was presented first it was judged as more cute than when it was shown second. Another possible explanation for the observed order effects of Study 1 is demand effects. The experimental videos combined with the subsequent cuteness scale might have tended to make participants feel that it would be socially undesirable to rate an animal as “not at all” cute. Given that in Study 1, the effect of the experimental manipulation was significant in both order conditions, and in Study 2 there was no interaction between order and condition, the order effects do not invalidate our conclusions.

Correlations between the trait empathic concern scale and the kama muta components could possibly be due to an artifact: common method variance resulting from individual differences in willingness to report tender, caring emotions. We found gender differences in levels of kama muta component ratings and cuteness ratings (see Supplementary Material), which may partially or completely result from correlated gender differences in disposition to report the emotion, and to report judgments that the stimuli are cute. So there is a possibility that responses to the IRI empathic concern trait subscale, the KAMMUS, and the cuteness items share variance due to individual differences in social desirability or impression management with regard to revealing, or even acknowledging to oneself, feelings and judgments judged to be feminine, juvenile, or embarrassing. If so, such shared method variance may contribute to the observed correlations among the measures.

Another limitation of the current studies concerns the data collection and data quality. The use of convenience sampling and relatively high drop-out and exclusion rates do not threaten the internal validity (as the experimental conditions were manipulated within participants and fully randomized), but they do suggest that the sample may not have been representative on relevant dimensions of the Norwegian population especially. For example, people sensitive to cuteness may have been more likely to actually complete the whole study. This was less of a problem for the United States sample in Study 1, in which participants were paid for their time. In this light, the convergence of the findings for Norway and the United States bolsters the central conclusions.

A statistical issue in the current studies was the high skew of some of the measures. For example, the sensations and signs of tears and goosebumps were rarely reported, skewing the distributions. To check the robustness of the findings for such measures, these analyses were therefore repeated using non-parametric models. The main results did not differ substantially from the multilevel models (see Supplementary Tables 15, 17). Thus, this problem does not appear to invalidate the obtained findings.

### Implications and Directions for Future Research

#### Implications for Emotion Research

The current studies have implications for theories of emotions in general and for emotional constructs similar to kama muta in particular. The evidence that 100s of participants report being *moved* by cute videos seems difficult to reconcile with Cova and Deonna (2014) and Deonna (2018) claim that *being moved* consists of the experience of a positive, transcendentally significant core value. They write that *being moved* (or *être ému*) “is the experience of a positive core value... perceived by the moved subject as standing out” (Cova and Deonna, 2014, p. 447). They continue, “‘Core values’ may be said to be those that a moral community treats as possessing ‘transcendental significance’ which preclude comparisons, trade-offs, or indeed any mingling with more mundane values” (see also Deonna, 2018). This conceptualization appears to preclude participants reporting that they are *moved* by cute kittens and puppies.

Likewise, Haidt (2000; Algoe and Haidt, 2009) theorizes that the emotion of *elevation* occurs as a result of observing or hearing about “moral beauty” or acts that reveal “humanity’s higher or better nature.” Haidt (2003, p. 281) points out that “the popular press and Oprah Winfrey talk about it (as being touched, moved, or inspired).” He characterizes elevation as involving a feeling of opening up and merging with others, and being motivated to help others. Haidt (2003, p. 282) indicates that elevation is recognizable by the “warm or glowing feeling in the chest,” along with “tingling.” There are many measures of elevation, but most of them include ratings of *being moved*, while many include sensations and signs such as warmth in the chest, a lump in the throat, and goosebumps or chills (Pohling and Diessner, 2016; Thomson and Siegel, 2017; see Zickfeld et al., in press). These sensations and signs and labels are among the sensations and signs and labels that many previous studies have shown to characterize kama muta (e.g., Schubert et al., 2016; Seibt et al., 2018; Zickfeld et al., 2019). Thus, the elevation construct seems to overlap considerably with kama muta. To the extent that the emotion states posited by the elevation and kama muta theories are phenomenologically similar, it appears inconsistent with elevation theory to find that people report that they are *moved, touched*, or have warm sensations in the chest when they look at images or videos of cute kittens or puppies. Cute kittens and puppies, wonderful as they are, probably do not instantiate either moral beauty or humanity’s higher nature.

Finally, the evidence for a clear and definite *but unnamed* emotional response to cuteness appears inconsistent with definitions and theories that emotions consist of the labeling of sensations and signs (Cannon, 1927; Lang, 1994; Barrett, 2017). It is crucial to those theories that all emotional experiences have readily accessible lexical names; for these theories, a person must give a name to their sensations and signs, or else the person is not experiencing an emotion. Yet neither Americans nor Norwegians can readily name what it is they feel when they see something cute; they simply characterize the evocative target with an adjective such as *cute, adorable*, or *sweet*. Hence, our findings that Americans and Norwegians nevertheless do have a definite emotion in response to cuteness poses a challenge to the labeling-of-sensations and signs theories. In contrast to Norwegian or English, an emotional response to cuteness has a definite name in Hungarian, Finnish, Estonian, and Telugu. So it would be interesting to see whether kama muta responses to cuteness are stronger for speakers of these languages – perhaps labeling, while not essential, amplifies awareness, memory, and reporting of an emotion.

#### Future Directions: Investigating the Mechanism Behind Kama Muta as a Cuteness Response

Why do cute animals evoke kama muta? In one line of research, Kindchenschema facial features are thought to be adaptive because they motivate tender caretaking, empathy for, and protection of one’s own vulnerable, needy offspring (Lorenz, 1943; Bradshaw and Paul, 2010; Leitão and Castelo-Branco, 2010; Sherman and Haidt, 2011). Consistent with this, facial cuteness (Keating et al., 2003) and facial *vulnerability* (van de Ven et al., 2016) evoke similar helping-related behaviors. The Stereotype Content Model (SCM; Cuddy et al., 2007; Fiske, 2015) makes a conceptual connection between perceived vulnerability and care, proposing that perceived target warmth and low competence result in pity and sympathy that in turn elicits helping and protective behavior (Fiske, 2012). Signs of vulnerability—being easily harmed by external forces—include young age, small size, small weight, signs of fragility, and weakness, whose effects are enhanced by environmental cues of imminent danger (Dijker, 2014). Concomitantly, people tend to associate the Kindchenschema with fragility, physical weakness, naiveté, warmth, and kindness (Berry and McArthur, 1985).

However, in another line of research, Sherman and Haidt (2011) argue that cuteness is a social engagement response; Rather than cuteness only evoking parental caretaking motives, cuteness evokes engagement/affiliative motives (such as to talk to, or play with the cute entity). In line with this proposal, infants at the peak of their vulnerability were rated as less cute than 6 to 10-month-old babies (Hildebrandt and Fitzgerald, 1979; Sanefuji et al., 2007). Additionally, babies displaying negative emotions (such as crying) were rated as less cute than children displaying positive emotions (such as smiling; Hildebrandt, 1983). From this they conclude that, as 6 to 10-month-old babies are more social, and smiling babies express more sociality, it is human sociality that is motivated by the cuteness response, and not caretaking. Furthermore, Sherman and Haidt (2011) predict that cute agents are anthropomorphized as social connection is an important motivator for anthropomorphism (Epley et al., 2008).

The studies in the current paper were not designed to compare the vulnerability and the social engagement accounts, as the main focus was to show that kama muta in fact is evoked by cute agents. Kama muta theory claims that the emotion motivates persons to devote themselves to a CS relationship. Such a relationship is characterized by responding to the needs of the relationship partner and it is also intrinsically rewarding. Given that the needs of human infants include not only being fed and protected, but also playing and talking, all of these motivations are likely to be higher for cuter agents. Furthermore, as CS is an intrinsically motivating and enjoyable relation, persons should also experience joy when interacting with cute agents. Future studies should investigate the mechanism behind kama muta responses to cuteness by distinguishing more clearly between different motivations evoked by cuteness.

#### Other Future Directions

Future studies should also seek evidence that a kama muta response evoked by cuteness motivates people to extend care, help, and compassion to the targets or others. Cuteness is frequently linked to perceived vulnerability and distress (e.g., Gross, 1997; Nenkov and Scott, 2014), which is hypothesized to evoke pity and sympathy (Cuddy et al., 2007).

While the goal of these studies was to test whether kama muta is the emotion evoked by seeing cuteness, this was only tested with videos of animals. It remains to be shown whether the obtained results hold for other cute agents, notably human babies, children, some adults, and artistic creations such as cartoon characters.

A final direction for subsequent research goes into a clinical domain. Animal Assisted Therapy improves emotional well-being (Nimer and Lundahl, 2007). It would be interesting to see whether kama muta mediates this therapeutic effect. There are also programs that bring animals to visit hospital patients, and ones that bring animals to sooth students stressed by exams. It might be that the benefits of interaction with affectionate animals is due to people’s kama muta responses to them.

## Conclusion

Features such as large eyes, a small nose, facial features low on the head (leaving a high forehead), a round face, and a large head comprise the *Kindchenschema* or baby schema; people perceive this schema as cute. Such cute features are neotenous, meaning they are characteristic of infants and gradually diminish with maturation. Mammalian survival depends on parents’ Kindchenschema-induced motivation to nurture and protect their offspring. Yet this emotion has been little studied in humans. We postulated that a typical emotional response to cuteness is kama muta. Kama muta is evoked by a sudden intensification of a CS relationship, and often denoted in English as *being touched, moved*, or having a *heartwarming* experience. The present project further hypothesized that CS interactions would increase cuteness perceptions of cute animals, and that kama muta would mediate this effect. Two experimental studies provided strong experimental support for both hypotheses.

In sum, the evidence of kama muta responses to cute kittens and puppies poses intriguing challenges to existing understandings of emotions. If these experiments are not persuasive, one only needs to open a browser and search for “cute images and videos.” The enormous amount of cute content on the Internet, the number of views and likes, and the responses that people post in response to them provide overwhelming evidence for the ubiquity and impact of kama muta responses to cuteness.

## Data Availability

All datasets, stimulus material and procedures are available at our OSF project page: https://osf.io/bjuva/.

## Ethics Statement

The current research was carried out in accordance with the recommendations out forward by the Norwegian Centre for Research Data and the Declaration of Helsinki where all subjects gave informed consent. Both studies described in this article received ethical approval from the Department of Psychology’s Research Ethics Committee at the University of Oslo, Norway.

## Author Contributions

KS, BS, and AF designed the studies. KS produced the stimulus material, programmed, and conducted the studies. KS and BS wrote the introduction. JB and JZ analyzed the data, wrote up the methods and results, and created the tables, figures, and Supplementary Material. All authors contributed to the general discussion and edited the paper.

## Conflict of Interest Statement

The authors declare that the research was conducted in the absence of any commercial or financial relationships that could be construed as a potential conflict of interest.

## Footnotes

1 Information gathered from linguistic raw data compiled by Fiske in 2018–2019.

2 See for example: https://www.youtube.com/watch?v=D6r9cst8OMU; https://www.youtube.com/watch?v=PjmlmyI56-k. A keyword search among the 1.509 comments to the first video (as of Septemeber 3, 2018) shows 365 mentions of either “cute,” “sweet,” or “adorable” and only 1 of either “moved” or “touched” used as an emotional term, indicating that English speakers tend to spontaneously label the object of their cuteness response but not the response itself.

3 Recent research suggests that infantile sounds and smells are also components of the *Kindchenschema* (see Kringelbach et al., 2016). The current paper, however, uses the term “cuteness” only in reference to the visual characteristics of the Lorenzian Kindchenschema, simply because our experimental stimuli were purely visual.

4 https://osf.io/bjuva/

5 We also had three additional hypotheses regarding effects of gender, pet ownership, and number of children that are not focal to the present investigation; for those analyses, see the Supplementary Materials.

6 The survey was translated to Norwegian, then back-translated and reconciled (Brislin, 1970). Both versions were available for both samples, and participants chose whether they wanted to respond in English or Norwegian.

7 Note that the sample was included in previously published research to test a different research question (Zickfeld et al., 2017b). Results and sample characteristics might differ minimally due to different exclusion criteria.

8 We decided to discard three reverse scored items based on a factor analysis revealing that these items loaded on a separate factor (see Supplementary Materials for the factor analysis).

9 Only three items of the Labels scale and four items of the Motivation scale were utilized in the analyses, as a later validation of the KAMMUS scale found that only these particular items are necessary to measure kama muta labels and motivation (Zickfeld et al., 2019).

10 Most of our variables of interest showed skewed distributions. So we repeated the main analyses for each of the five DVs using a Wilcoxon signed rank test with condition as predictor. The results did not differ from the findings using multilevel models and can be found in the Supplementary Material.

11 For each model, we ran an initial test including nationality, video type, and video sound as additional predictors. We only observed a significant nationality^∗^order interaction for the cuteness and positivity models. In both cases the order effect was more pronounced for the United States participants.

12 While we observed a significant interaction effect between order and condition, testing our main models separately for first and second order effects revealed that all effects were in the predicted direction and their 95% CIs did not include zero.

13 We also pre-registered two additional hypotheses not focal to the present research question. All hypotheses and planned statistical analyses were preregistered at AsPredicted.com, 20^th^ September 2016.

14 Based on repeated ANOVAs rather than the mixed models used here.

15 Note that the sample was included in previously published research to test a different research question (Zickfeld et al., 2017b). Results and sample characteristics might differ minimally due to different exclusion criteria.

16 In videos showing a human, only the hands of the person were visible, in order to limit participants’ cuteness appraisals to the animal.

17 Most of our variables of interest showed skewed distributions. So we repeated the main analyses using a Wilcoxon signed rank test with condition as predictor for each of the seven DVs. The results did not differ from the findings using multilevel models; they can be found in the Supplementary Material.

18 Table 4 suggests an interaction effect between empathic concern and condition. We therefore repeated the main model including the interaction between these variables in an exploratory fashion. We did not find a statistically significant interaction effect in the full model, but did so when including only condition and empathic concern as predictors.

19 By use of the following website: https://msu.edu/~falkcarl/mediation.html.

## References

[B1] Algoe S. B. Haidt J. (2009). Witnessing excellence in action: the “other-praising” emotions of elevation, gratitude, and admiration. *J. Posit. Psychol.* 4 105–127. 10.1080/17439760802650519 19495425 PMC2689844

[B2] Aragón O. R. Bargh J. A. (2018). So happy i could shout!” and “so happy i could cry!” dimorphous expressions represent and communicate motivational aspects of positive emotions. *Cogn. Emot.* 32 286–302. 10.1080/02699931.2017.1301388 28415957

[B3] Aragón O. R. Clark M. S. Dyer R. L. Bargh J. A. (2015). Dimorphous expressions of positive emotion: displays of both care and aggression in response to cute stimuli. *Psychol. Sci.* 26 259–273. 10.1177/0956797614561044 25626441

[B4] Baron Z. (2014). *Where the Wild Things go Viral.* Available at: https://www.gq.com/story/buzzfeed-beastmaster-profile-march-2014 [accessed September 5, 2018].

[B5] Barrett L. F. (2017). *How Emotions are Made: The Secret Life of the Brain.* Boston: Houghton Mifflin Harcourt.

[B6] Batson C. D. Fultz J. Schoenrade P. A. (1987). Distress and empathy: two qualitatively distinct vicarious emotions with different motivational consequences. *J. Pers.* 55 19–39. 10.1111/j.1467-6494.1987.tb00426.x 3572705

[B7] Batson C. D. Lishner D. A. Cook J. Sawyer S. (2005). Similarity and nurturance: two possible sources of empathy for strangers. *Basic Appl. Soc. Psych.* 27 15–25. 10.1207/s15324834basp2701_2

[B8] Bauer D. J. Preacher K. J. Gil K. M. (2006). Conceptualizing and testing random indirect effects and moderated mediation in multilevel models: new procedures and recommendations. *Psychol. Methods* 11 142–163. 10.1037/1082-989X.11.2.142 16784335

[B9] Bellfield J. Bimont C. Blom J. Dommeyer C. J. Gardiner K. Mathenia E. (2011). The effect of a cute stimulus on personally-Initiated, self-adminstered surveys. *Mark. Bull.* 22 1–9.

[B10] Berry D. S. McArthur L. Z. (1985). Some components and consequences of a babyface. *J. Pers. Soc. Psychol.* 48 312–323. 10.1037/0022-3514.48.2.312

[B11] Borgi M. Cogliati-Dezza I. Brelsford V. Meints K. Cirulli F. (2014). Baby schema in human and animal faces induces cuteness perception and gaze allocation in children. *Front. Psychol.* 5:411. 10.3389/fpsyg.2014.00411 24847305 PMC4019884

[B12] Bowman N. A. (2012). Effect sizes and statistical methods for meta-analysis in higher education. *Res. High. Educ.* 53 375–382. 10.1007/s11162-011-9232-5

[B13] Bradshaw J. W. S. Paul E. S. (2010). Could empathy for animals have been an adaptation in the evolution of homo? *Anim. Welf.* 19 107–112.

[B14] Brislin R. W. (1970). Back-translation for cross-cultural research. *J. Cross. Cult. Psychol.* 1 185–216. 10.1177/135910457000100301

[B15] Buckley R. C. (2016). Aww: the emotion of perceiving cuteness. *Front. Psychol.* 7:1740. 10.3389/fpsyg.2016.01740 27891103 PMC5102905

[B16] Cannon W. B. (1927). The james-lange theory of emotions: a critical examination and an alternative theory. *Am. J. Psychol.* 39 106–124. 10.2307/1415404 3322057

[B17] Cova F. Deonna J. A. (2014). Being moved. *Philos. Stud.* 169 447–466. 10.1007/s11098-013-0192-9

[B18] Cuddy A. J. C. Fiske S. T. Glick P. (2007). The BIAS map: behaviors from intergroup affect and stereotypes. *J. Pers. Soc. Psychol.* 92 631–648. 10.1037/0022-3514.92.4.631 17469949

[B19] Dale J. P. (2016). Cute studies: an emerging field. *East Asian J. Pop. Cult.* 2 5–13. 10.1386/eapc.2.1.5_2

[B20] Davis M. H. (1980). A multidimensional approach to individual differences in emphaty. *Cat. Sel. Doc. Psychol.* 10:85.

[B21] Davis M. H. (1983). Measuring individual differences in empathy: evidence for a multidimensional approach. *J. Pers. Soc. Psychol.* 44 113–126. 10.1037/0022-3514.44.1.113

[B22] Deonna J. A. (2018). “The emotion of being moved,” in *Shadows of the Soul: Philosophical Perspectives on Negative Emotions*, eds Tappolet C. Teroni F. Ziv A. K. (New York, NY: Routledge), 60–68. 10.4324/9781315537467-7

[B23] Dijker A. J. M. (2014). A theory of vulnerability-based morality. *Emot. Rev.* 6 175–183. 10.1177/1754073913514120

[B24] Duffy S. A. Burton D. (2000). Cartoon characters as tobacco warning labels. *Arch. Pediatr. Adolesc. Med.* 154 1230–1236. 10.1001/archpedi.154.12.1230 11115308

[B25] Epley N. Akalis S. Waytz A. Cacioppo J. T. (2008). Creating social connection through inferential reproduction. *Psychol. Sci.* 19 114–120. 10.1111/j.1467-9280.2008.02056.x 18271858

[B26] Falk C. F. Biesanz J. C. (2016). Two cross-platform programs for inferences and interval estimation about indirect effects in mediational models. *SAGE Open J.* 6:2158244015625445 10.1177/2158244015625445

[B27] Fiske A. P. (1991). *Structures of Social Life: The Four Elementary Forms of Human Relations.* New York, NY: Free Press.

[B28] Fiske A. P. (1992). The four elementary forms of sociality: framework for a unified theory of social relations. *Psychol. Rev.* 99 689–723. 10.1037/0033-295X.99.4.689 1454904

[B29] Fiske A. P. (2004). “Four modes of constituting relationships: consubstantial assimilation; space, magnitude, time, and force; concrete procedures; abstract symbolism,” in *Relational Models Theory: A Contemporary Overview*, ed. Haslam N. (Mahwah, NJ: Erlbaum), 61–146.

[B30] Fiske A. P. Schubert T. W. Seibt B. (2017a). “‘kama muta’ or ‘being moved by love’: a bootstrapping approach to the ontology and epistemology of an emotion,” in *Universalism Without Uniformity: Explorations in Mind and Culture*, eds Cassaniti J. Menon U. (Chicago: University of Chicago Press), 79–100. 10.7208/chicago/9780226501710.001.0001

[B31] Fiske A. P. Schubert T. W. Seibt B. (2017b). The best loved story of all time: overcoming all obstacles to be reunited, evoking kama muta. *Evol. Stud. Imaginat. Cult.* 1 67–70. 10.26613/esic/1.1.12

[B32] Fiske A. P. Seibt B. Schubert T. W. (2017c). The sudden devotion emotion: kama muta and the cultural practices whose function is to evoke it. *Emot. Rev.* 11 74–86. 10.1177/1754073917723167

[B33] Fiske S. T. (2012). Warmth and competence: stereotype content issues for clinicians and researchers. *Can. Psychol. Can.* 53 14–20. 10.1037/a0026054 24155504 PMC3801417

[B34] Fiske S. T. (2015). Intergroup biases: a focus on stereotype content. *Curr. Opin. Behav. Sci.* 3 45–50. 10.1016/J.COBEHA.2015.01.010 27453920 PMC4955357

[B35] Glocker M. L. Langleben D. D. Ruparel K. Loughead J. W. Gur R. C. Sachser N. (2009). Baby schema in infant faces induces cuteness perception and motivation for caretaking in adults. *Ethology* 115 257–263. 10.1111/j.1439-0310.2008.01603.x 22267884 PMC3260535

[B36] Golle J. Lisibach S. Mast F. W. Lobmaier J. S. (2013). Sweet puppies and cute babies: perceptual adaptation to babyfacedness transfers across species. *PLoS One* 8:e58248. 10.1371/journal.pone.0058248 23516453 PMC3596402

[B37] Gould S. J. (1980). *A Biological Homeage to Micket Mouse. The Panda’s Thumb?: More Reflections in Natural History.* New York, NY: W. W. Norton & Company, 95–107.

[B38] Gross T. F. (1997). Children’s perception of faces of varied immaturity. *J. Exp. Child Psychol.* 66 42–63. 10.1006/JECP.1997.2373 9226933

[B39] Haidt J. (2000). The positive emotion of elevation. *Prev. Treat.* 3. 10.1037/1522-3736.3.1.33c

[B40] Haidt J. (2003). “Elevation and the positive psychology of morality,” in *Flourishing: Positive Psychology and the Life Well-Lived*, eds Keyes C. L. M. Haidt J. (Washington, DC: American Psychological Association), 275–289. 10.1037/10594-012

[B41] Haslam N. (2006). Dehumanization?: an integrative review. *Pers. Soc. Psychol. Rev.* 10 252–264. 10.1207/s15327957pspr1003_4 16859440

[B42] Haslam N. (2017). *Goosebumps, Tears and Tenderness: What it Means to be Moved.* Available at: https://theconversation.com/goosebumps-tears-and-tenderness-what-it-means-to-be-moved-72545 [accessed September 5, 2018].

[B43] Hildebrandt K. A. (1983). Effect of facial expression variations on ratings of infants’ physical attractiveness. *Dev. Psychol.* 19 414–417. 10.1037/0012-1649.19.3.414 855947

[B44] Hildebrandt K. A. Fitzgerald H. E. (1978). Adults’ responses to infants varying in perceived cuteness. *Behav. Process.* 3 159–172. 10.1016/0376-6357(78)90042-6 24924654

[B45] Hildebrandt K. A. Fitzgerald H. E. (1979). Facial feature determinants of perceived infant attractiveness. *Infant Behav. Dev.* 2 329–339. 10.1016/S0163-6383(79)80043-0

[B46] Hildebrandt K. A. Fitzgerald H. E. (1981). Mothers’ responses to infant physical appearance. *Infant Ment. Health J.* 2 56–61. 10.1002/1097-0355(198121)2:1<56::AID-IMHJ2280020109>3.0.CO;2-G

[B47] Hodson G. MacInnis C. C. Costello K. (2015). “(Over)valuing ‘humanness’ as an aggravator of intergroup prejudices and discrimination,” in *Humanness and Dehumanization*, eds Bain P. G. Vaes J. Leyens J. (New York, NY: Psychology Press), 89–110.

[B48] Huddy L. Gunnthorsdottir A. H. (2000). The persuasive effects of emotive visual imagery: superficial manipulation or the product of passionate reason? *Polit. Psychol.* 21 745–778. 10.1111/0162-895X.00215 17645126

[B49] Keating C. F. Randall D. W. Kendrick T. Gutshall K. A. (2003). Do babyfaced adults receive more help? The (cross-cultural) case of the lost resume. *J. Nonverbal. Behav.* 27 89–109. 10.1023/A:1023962425692

[B50] Kringelbach M. L. Stark E. A. Alexander C. Bornstein M. H. Stein A. (2016). On cuteness: unlocking the parental brain and beyond. *Trends Cogn. Sci.* 20 545–558. 10.1016/J.TICS.2016.05.003 27211583 PMC4956347

[B51] Labato R. Meese J. (2014). Kittens all the way down: cute in context. *M/C J.* 17.

[B52] Lang P. J. (1994). The varieties of emotional experience: a meditation on james-lange theory. *Psychol. Rev.* 101 211–221. 10.1037/0033-295X.101.2.211 8022956

[B53] Lang P. J. Bradley M. M. Cuthbert B. N. (1997). *International Affective Picture System (IAPS): Technical Manual and Affective Ratings.* Gainesville, FL: NIMH Center for the Study of Emotion and Attention, 39–58.

[B54] Lehmann V. Huis in’t Veld E. M. Vingerhoets A. J. (2013). The human and animal baby schema effect: correlates of individual differences. *Behav. Process.* 94 99–108. 10.1016/j.beproc.2013.01.001 23353724

[B55] Leitão M. Castelo-Branco R. (2010). Babies: the irresistible power of cuteness. A study concerning the evolutionary function of infantile traits. *Estud. Psicol.* 15 71–78. 10.1590/S1413-294X2010000100010

[B56] Levin J. Arluke A. Irvine L. (2017). Are people more disturbed by dog or human suffering? *Soc. Anim.* 25 1–16. 10.1163/15685306-12341440

[B57] Lishner D. A. Oceja L. V. Stocks E. L. Zaspel K. (2008). The effect of infant-like characteristics on empathic concern for adults in need. *Motiv. Emot.* 32 270–277. 10.1007/s11031-008-9101-5

[B58] Little A. C. (2012). Manipulation of infant-like traits affects perceived cuteness of infant, adult and cat faces. *Ethology* 118 775–782. 10.1111/j.1439-0310.2012.02068.x

[B59] Lorenz K. (1943). Die angeborenen formen möglicher erfahrung. *Z. Tierpsychol.* 5 235–409. 10.1111/j.1439-0310.1943.tb00655.x

[B60] McDougall W. (1919). *An Introduction to Social Psychology*, 14th Edn. London: Methuen.

[B61] McDougall W. (1923). *Outline of Psychology.* New York, NY: Charles Scribner’s Sons.

[B62] Moors A. Ellsworth P. C. Scherer K. R. Frijda N. H. (2013). Appraisal theories of emotion: state of the art and future development. *Emot. Rev.* 5 119–124. 10.1177/1754073912468165

[B63] Morris P. H. Reddy V. Bunting R. C. (1995). The survival of the cutest: who’s responsible for the evolution of the teddy bear? *Anim. Behav.* 50 1697–1700. 10.1016/0003-3472(95)80022-0

[B64] Nenkov G. Y. Scott M. L. (2014). “So cute i could eat it up”: priming effects of cute products on indulgent consumption. *J. Consum. Res.* 41 326–341. 10.1086/676581

[B65] Niezink L. W. Siero F. W. Dijkstra P. Buunk A. P. Barelds D. P. H. (2012). Empathic concern: distinguishing between tenderness and sympathy. *Motiv. Emot.* 36 544–549. 10.1007/s11031-011-9276-z 23144514 PMC3491184

[B66] Nimer J. Lundahl B. (2007). Animal-assisted therapy: a meta-analysis. *Anthrozoos* 20 225–238. 10.2752/089279307X224773

[B67] Nittono H. (2016). The two-layer model of “kawaii”: a behavioural science framework for understanding kawaii and cuteness. *East Asian J. Pop. Cult.* 2 79–95. 10.1386/eapc.2.1.79_1

[B68] Nittono H. Fukushima M. Yano A. Moriya H. (2012). The power of kawaii: viewing cute images promotes a careful behavior and narrows attentional focus. *PLoS One* 7:e46362. 10.1371/journal.pone.0046362 23050022 PMC3458879

[B69] Nittono H. Ihara N. (2017). Psychophysiological responses to kawaii pictures with or without baby schema. *SAGE Open* 7 1–11. 10.1177/2158244017709321

[B70] Opotow S. (1990). Moral exclusion and injustice: an introduction. *J. Soc. Issues* 46 1–20. 10.1111/j.1540-4560.1990.tb00268.x

[B71] Pittenger J. B. (1990). Body proportions as information for age and cuteness: animals in illustrated children’s books. *Percept. Psychophys.* 48 124–130. 10.3758/BF03207078 2385485

[B72] Pohling R. Diessner R. (2016). Moral elevation and moral beauty: a review of the empirical literature. *Rev. Gen. Psychol.* 20 412–425. 10.1037/gpr0000089

[B73] Ruanguttamanun C. (2014). The use of appeals in green printed advertisements: a case of product orientation and organizational image orientation ads. *Int. J. Humanit. Soc. Sci.* 8 2988–2993.

[B74] Sanefuji W. Ohgami H. Hashiya K. (2007). Development of preference for baby faces across species in humans (homo sapiens). *J. Ethol.* 25 249–254. 10.1007/s10164-006-0018-8 28406237

[B75] Schubert T. W. Waldzus S. Seibt B. (2008). “The embodiment of power and communalism in space and bodily contact,” in *Embodied Grounding: Social, Cognitive, Affective, and Neuroscientific Approaches*, eds Semin G. R. Smith E. R. (Cambridge: Cambridge University Press), 160–183.

[B76] Schubert T. W. Zickfeld J. H. Seibt B. Fiske A. P. (2016). Moment-to-moment changes in feeling moved match changes in closeness, tears, goosebumps, and warmth: time series analyses. *Cogn. Emot.* 32 174–184. 10.1080/02699931.2016.1268998 28024440

[B77] Seibt B. Schubert T. W. Zickfeld J. H. Fiske A. P. (2017a). Interpersonal closeness and morality predict feelings of being moved. *Emotion* 17 389–394. 10.1037/emo0000271 28150953

[B78] Seibt B. Schubert T. W. Zickfeld J. H. Zhu L. Arriaga P. Simão C. (2017b). Kama muta: similar emotional responses to touching videos across the US, Norway, China, Israel, and Portugal. *J. Cross Cult. Psychol.* 49 418–435. 10.1177/0022022117746240

[B79] Seibt B. Schubert T. W. Zickfeld J. H. Fiske A. P. (2018). Touching the base: heart-warming ads from the 2016 U.S. election moved viewers to partisan tears. *Cogn. Emot.* 10.1080/02699931.2018.1441128 [Epub ahead of print]. 29510656

[B80] Sherman G. D. Haidt J. (2011). Cuteness and disgust: the humanizing and dehumanizing effects of emotion. *Emot. Rev.* 3 245–251. 10.1177/1754073911402396

[B81] Sherman G. D. Haidt J. Iyer R. Coan J. A. (2013). Individual differences in the physical embodiment of care: prosocially oriented women respond to cuteness by becoming more physically careful. *Emotion* 13 151–158. 10.1037/a0029259 22889413

[B82] Simmons J. P. Nelson L. D. Simonsohn U. (2011). False-positive psychology: undisclosed flexibility in data collection and analysis allows presenting anything as significant. *Psychol. Sci.* 22 1359–1366. 10.1177/0956797611417632 22006061

[B83] Thomson A. L. Siegel J. T. (2017). Elevation: a review of scholarship on a moral and other-praising emotion. *J. Posit. Psychol.* 12 628–638. 10.1080/17439760.2016.1269184

[B84] Urban Dictionary (2008). *Cuteness Overload.* Available at: https://bit.ly/2XoaAUv [accessed September 13, 2018].

[B85] Urban Dictionary (2009). *Cute Attack.* Available at: https://bit.ly/2ViELKU [accessed September 13, 2018].

[B86] van de Ven N. Meijs M. H. J. Vingerhoets A. (2016). What emotional tears convey: tearful individuals are seen as warmer, but also as less competent. *Br. J. Soc. Psychol.* 56 146–160. 10.1111/bjso.12162 27709633 PMC5363367

[B87] Volk A. A. Lukjanczuk J. L. Quinsey V. L. (2007). Perceptions of child facial cues as a function of child age. *Evol. Psychol.* 5 801–814. 10.1177/147470490700500409

[B88] Zickfeld J. H. (2015). *Heartwarming Closeness. Being Moved Induces Communal Sharing and Increases Feelings of Warmth.* Available at: http://urn.nb.no/URN:NBN:no-52508

[B89] Zickfeld J. H. Kunst J. R. Hohle S. M. (2017a). Too sweet to eat: exploring the effects of cuteness on meat consumption. *Appetite* 120 181–195. 10.1016/J.APPET.2017.08.038 28882424

[B90] Zickfeld J. H. Schubert T. W. Seibt B. Fiske A. P. (2017b). Empathic concern is part of a more general communal emotion. *Front. Psychol.* 8:723. 10.3389/fpsyg.2017.00723 28539901 PMC5423947

[B91] Zickfeld J. H. Schubert T. W. Seibt B. Blomster J. K. Arriaga P. Basabe N. (2019). Kama muta: conceptualizing and measuring the experience often labelled *being moved* across 19 nations and 15 languages. *Emotion.* 10.1037/emo0000450 [Epub ahead of print]. 29888936

[B92] Zickfeld J. H. Schubert T. W. Seibt B. Fiske A. P. (in press). Moving through the literature: what is the emotion often denoted being moved? *Emot. Rev.* 10.31234/osf.io/pndce

