# Supplementary material for: Too Cute for Words: Cuteness Evokes the Heartwarming Emotion of Kama Muta
Source: Front Psychol. 2019 Mar 1;10:387. doi: 10.3389/fpsyg.2019.00387 (PMC6405428; doi:10.3389/fpsyg.2019.00387)
Supplement: Supplementary file 1 [file Table_1.DOCX]

Supplementary Material

Too Cute for Words:
Cuteness Evokes the Heartwarming Emotion of Kama Muta

Kamilla Knutsen Steinnes*^†^, Johanna Katarina Blomster^†^, Beate Seibt, Janis H. Zickfeld, Alan Page Fiske

*** Correspondence:** Kamilla Knutsen Steinnes: [kamillak@oslomet.no](mailto:kamillak@oslomet.no)

^†^ These authors have shared first authorship and contributed equally to this work.

# Scales used in both studies

## KAMMUS version 1.8 used in Study 1

In all sections, Likert scales from 0 = *not at all* to 6 = *a lot* were used. Sections were presented in the order indicated; items were randomized within each section.

Supplementary Table 1. Section 1: Sensations and Signs

| Item | English | Norwegian | M(SD) | Skew/ Kurtosis |
| --- | --- | --- | --- | --- |
| 1 | Moist eyes | Fuktige (tårevåte) øyne | .28(.92) | 4.12/18.30 |
| 2 | Tears | Tårer | .12(.55) | 6.16/46.12 |
| 3 | Goosebumps or hair standing up | Gåsehud eller hår reiser seg | .30(.84) | 3.40/12.57 |
| 4 | Chills or shivers | Frysninger eller skjelvinger | .38(1.03) | 3.13/10.05 |
| 5 | A warm feeling in the center of the chest | En varm følelse i midten av brystet | 1.09(1.70) | 1.38/.63 |
| 6 | A swelling or tingling feeling in the center of the chest | En svulmende eller prikkende følelse i midten av brystet | .44(1.12) | 2.91/8.29 |
| 7 | Choked up or a lump in the throat | En klump i halsen | .19(.67) | 4.66/25.75 |
| 8 | I put one or both hands to my chest | Jeg la en eller begge hender på brystet | .27(.90) | 4.15/19.03 |
| 9 | I took a deep breath or held my breath | Jeg tok et dypt åndedrag eller holdt pusten | .45(1.15) | 2.86/8.00 |
| 10 | I said something like “awww” | Jeg sa noe slikt som ”tnååå” | 1.45(2.12) | 1.10/-.37 |
| 11 | Buoyant or light | Svevende eller lett | 1.45(1.80) | .91/-.42 |
| 12 | Refreshed, energized, or exhilarated | Forfrisket, energisk eller oppkvikket | 1.49(1.78) | .84/-.59 |

**Supplementary Table 2.** Section 3 & 4: Motivation for communal sharing relations

| Item | English | Norwegian | M(SD) | Skew/ Kurtosis |
| --- | --- | --- | --- | --- |
|  |  | ***Section 3*** |  |  |
| 1 | I felt like telling someone how much I care about them | Jeg følte for å fortelle noen hvor mye jeg bryr meg om dem | .71(1.30) | 1.95/3.28 |
| 2 | I wanted to hug someone | Jeg hadde lyst til å gi noen en klem | .94(1.53) | 1.54/1.25 |
| 3 | I wanted to do something extra-nice for someone | Jeg hadde lyst til å gjøre noe kjempesnilt for noen | .77(1.38) | 1.80/2.42 |
| 4 | I felt especially friendly toward nearly everyone | Jeg følte meg spesielt vennlig mot nesten alle andre | .89(1.45) | 1.60/1.65 |
| 5 | I felt more strongly committed to a relationship | Jeg følte en sterkere forpliktelse til et forhold | .63(1.28) | 2.19/4.21 |
|  |  | ***Section 4*** |  |  |
| 1 | I was eager to tell my friends or family about the experience | Jeg var ivrig etter å fortelle vennene mine eller familien min om opplevelsen | .90(1.53) | 1.78/2.29 |
| 2 | I wanted to have the experience all over again together *with* others | Jeg ønsket å ha opplevelsen om igjen *sammen* med andre | 1.02(1.58) | 1.48/1.22 |

**Supplementary Table 3.** Section 5: Emotional valence & labels

| Item | English | Norwegian | M(SD) | Skew/ Kurtosis |
| --- | --- | --- | --- | --- |
|  |  | ***Valence*** |  |  |
| 1 | I had positive feelings | Jeg hadde positive følelser | 3.01(2.14) | -.11/-1.32 |
| 2 | I had negative feelings | Jeg hadde negative følelser | .93(1.50) | 1.54/1.35 |
|  |  | ***Labels*** |  |  |
| 1 | It was heartwarming | Det var hjertevarmende | 1.70(2.03) | .78/-.85 |
| 2 | I was moved | Jeg var beveget | 1.06(1.60) | 1.42/.94 |
| 3 | I was touched | Jeg var rørt | 1.09(1.63) | 1.34/.59 |
| 4 | It was a nostalgic moment | Det var et nostalgisk øyeblikk |  |  |
| 5 | It was a poignant experience | Det var en gripende opplevelse |  |  |
| 6 | I identified with something larger than myself | Jeg følte meg som en del av noe større enn meg selv |  |  |

## KAMMUS version 2.0 used in Study 2

**Supplementary Table 4.** Section 1: Sensations and Signs

| Item | English | Norwegian | M(SD) | Skew/ Kurtosis |
| --- | --- | --- | --- | --- |
| 1 | Moist eyes | Fuktige (tårevåte) øyne | .36(1.07) | 3.36/11.07 |
| 2 | Tears | Tårer | .13(.66) | 6.60/48.67 |
| 3 | Goosebumps or hair standing up | Gåsehud eller hår reiser seg | .26(.89) | 4.17/18.63 |
| 4 | Chills or shivers | Frysninger eller skjelvinger | .21(.79) | 4.34/20.17 |
| 5 | A warm feeling in the center of the chest | En varm følelse i midten av brystet | 2.08(1.96) | .41/-1.13 |
| 6 | Some feeling in the center of the chest | En følelse i midten av brystet | 1.73(1.92) | .66/-.91 |
| 7 | Choked up | Gråtkvalt/tårekvalt | .15(.68) | 5.96/39.56 |
| 8 | A lump in the throat | En klump i halsen | .29(.91) | 3.58/13.11 |
| 9 | Difficulty speaking | Vanskelig for å snakke | .43(1.10) | 2.83/7.87 |
| 10 | I put one or both hands to my chest | Jeg la en eller begge hender på brystet | .50(1.35) | 2.85/7.14 |
| 11 | I took a deep breath or held my breath | Jeg tok et dypt åndedrag eller holdt pusten | .90(1.65) | 1.68/1.52 |
| 12 | I said something like “awww” | Jeg sa noe slikt som ”tnååå” | 2.22(2.19) | .46/-1.25 |
| 13 | Buoyant or light | Svevende eller lett | 1.78(1.78) | .66/-.63 |
| 14 | Refreshed, energized, or exhilarated | Forfrisket, energisk eller oppkvikket | 1.74(1.74) | .72/-.48 |

**Supplementary Table 5.** Section 2: Communal sharing sudden intensification appraisals

| Item | English "I observed…" | Norwegian "Jeg observerte…" | M(SD) | Skew/ Kurtosis |
| --- | --- | --- | --- | --- |
| 1 | ... an incredible bond | ... et utrolig bånd | 2.40(2.02) | .27/-1.23 |
| 2 | ... a special sense of belonging | ... en spesiell følelse av tilhørighet | 1.98(1.99) | .55/-1.08 |
| 3 | ... an exceptional sense of closeness appear | ... en utrolig følelse av nærhet oppstod | 2.15(1.94) | .46/-.99 |
| 4 | ... the emergence of a remarkable feeling of oneness | ... at en spesiell følelse av enhet oppstod | 1.42(1.74) | 1.02/-.10 |
| 5 | ... a unique kind of love spring up | ... at en unik type kjærlighet oppstod | 2.11(1.96) | .47/-1.01 |
| 6 | ... a phenomenal feeling of being appreciated | ... en utrolig følelse av å bli verdsatt | 1.67(1.90) | .80/-.60 |
| 7 | ... an astonishing sense of being needed | ... en utrolig følelse av å være ønsket | 1.80(1.91) | .65/-.88 |
| 8 | ... an extraordinary feeling of being welcomed | ... en fantastisk følelse av å være velkommen | 1.74(1.91) | .69/-.89 |
| 9 | ... exceptional care being given to someone | ... at eksepsjonell omsorg ble gitt | 2.45(2.05) | .31/-1.22 |
| 10 | ... a great kindness | ... en kjempesnill handling | 2.07(2.01) | .57/-.91 |

**Supplementary Table 6.** Section 3 & 4: Motivation for communal sharing relations

| Item | English | Norwegian | M(SD) | Skew/ Kurtosis |
| --- | --- | --- | --- | --- |
|  |  | ***Section 3*** |  |  |
| 1 | I felt like telling someone how much I care about them | Jeg følte for å fortelle noen hvor mye jeg bryr meg om dem | 1.50(1.77) | .98/-.09 |
| 2 | I wanted to hug someone | Jeg hadde lyst til å gi noen en klem | 2.13(2.02) | .48/-1.07 |
| 3 | I wanted to do something extra-nice for someone | Jeg hadde lyst til å gjøre noe kjempesnilt for noen | 1.67(1.80) | .80/-.47 |
| 4 | I felt especially friendly | Jeg følte meg spesielt vennlig | 1.78(1.78) | .73/-.49 |
| 5 | I felt more strongly committed to a relationship | Jeg følte en sterkere forpliktelse til et forhold | 1.25(1.73) | 1.27/.57 |
|  |  | ***Section 4*** |  |  |
| 1 | I was eager to tell my friends or family about the experience | Jeg var ivrig etter å fortelle vennene mine eller familien min om opplevelsen | .90(1.48) | 1.73/2.33 |
| 2 | I wanted to have the experience together *with* others | Jeg ønsket å ha opplevelsen *sammen* med andre | 1.77(1.97) | .76/-.76 |

**Supplementary Table 7.** Section 5: Emotional valence & labels

| Item | English | Norwegian | M(SD) | Skew/ Kurtosis |
| --- | --- | --- | --- | --- |
|  |  | ***Valence*** |  |  |
| 1 | I had positive feelings | Jeg hadde positive følelser | 3.88(1.77) | -.58/-.62 |
| 2 | I had negative feelings | Jeg hadde negative følelser | .78(1.30) | 1.81/2.76 |
|  |  | ***Labels*** |  |  |
| 1 | It was heartwarming | Det var hjertevarmende | 2.96(2.02) | -.01/-1.29 |
| 2 | I was moved | Jeg var beveget | 1.51(1.78) | .98/-.11 |
| 3 | I was touched | Jeg var rørt | 1.38(1.74) | 1.09/.10 |
| 4 | It was a nostalgic moment | Det var et nostalgisk øyeblikk | 1.08(1.70) | 1.45/.87 |
| 5 | It was a poignant experience | Det var en gripende opplevelse | 1.18(1.59) | 1.30/.91 |
| 6 | I felt a part of something larger than myself | Jeg følte meg som en del av noe større enn meg selv | .87(1.50) | 1.75/2.20 |
| 7 | I felt in love | Jeg følte meg forelsket | .80(1.47) | 1.98/3.23 |

In all sections, participants responded on Likert scales, 0 = *not at all* to 6 = *a lot*. Sections were presented to participants in the order indicated above; items were individually randomized within each section.

## Cuteness Scale

**Supplementary Table 8.** Cuteness Scale used in Study 1

| Item | English | Norwegian | M(SD) | Skew/ Kurtosis |
| --- | --- | --- | --- | --- |
| 1 | It is adorable | Det er bedårende | 2.92(2.40) | -.04/-1.63 |
| 2 | It is repulsive [R] | Det er frastøtende | 5.03(1.66) | -1.69/1.78 |
| 3 | It is sweet | Det er elskverdig | 2.79(2.37) | .03/-1.63 |
| 4 | It is huge [R] | Det er kjempestort | 4.62(1.77) | -1.08/.02 |
| 5 | It looks so soft | Det ser veldig mykt ut | 3.04(2.42) | -.11/-1.63 |
| 6 | It looks really old [R] | Det ser veldig gammelt ut | 4.84(1.65) | -1.37/.86 |
| 7 | It is cute | Det er søtt | 3.04(2.44) | -.10/-1.66 |
| 8 | It is cuddly | Det er veldig kosete | 2.66(2.50) | .12/-1.72 |
| 9 | I would like to pet it | Jeg har lyst å klappe det | 2.61(2.45) | .20/-1.63 |
| A | It is amusing [D] | Det er underholdende | 2.10(1.90) | .39/-1.10 |
| B | It is interesting [D] | Det er interessant | 3.54(1.96) | -.39/-1.03 |
| C | It is funny [D] | Det er morsomt | 2.00(1.86) | .52/-.84 |

Responses were recorded on a Likert scale, from 0 = *not at all* to 6 = *a lot.* [D] Distractor Item; [R] Reversed Item

**Supplementary Table 9.** Revised Cuteness Scale used in Study 2

| Item | English | Norwegian | M(SD) | Skew/ Kurtosis |
| --- | --- | --- | --- | --- |
| 1 | It is adorable | Det er bedårende | 3.68(1.77) | -.58/-.58 |
| 2 | It is not sweet [R] | Det er ikke elskverdig | 4.72(1.59) | -1.25/.80 |
| 3 | It looks so soft | Det ser så mykt ut | 5.08(1.32) | -1.92/3.79 |
| 4 | It is not cute [R] | Det er ikke søtt | 4.99(1.54) | -1.69/2.03 |
| 5 | It is cuddly | Det er veldig kosete | 4.00(1.86) | -.71/-.59 |
| 6 | I would not like to pet it [R] | Jeg ville ikke likt å klappe det | 4.45(2.26) | -1.08/-.55 |
| A | It is amusing [D] | Det er underholdende | 2.36(1.74) | .28/-.84 |
| B | It is not interesting [D] | Det er ikke interessant | 2.64(2.01) | .19/-1.21 |
| C | It is funny [D] | Det er morsomt | 1.60(1.61) | .85/-.11 |

Responses were recorded on a Likert scale, from 0 = *not at all* to 6 = *a lot.* [D] Distractor Item; [R] Reversed Item

# Video stimuli

## Study 1 Pretest of Video Stimuli

Sixteen YouTube video clips, eight in each condition, were pretested in a between-subjects design (N = 8) on mean cuteness-ratings of a single item (“How cute is this animal to you?”). Responses were given on a 7-point Likert scale, ranging from 0 (not cute at all) to 6 = (very cute). Four of the highest (in the experimental group) and lowest ranking videos (in the control group) were selected as the stimuli for study 1 (see Supplementary Table A for video links and means). The videos in the experimental condition featured young cute animals (e.g., a kitten), while the control condition featured adult, non-cute animals (e.g., a proboscis monkey). Each video clip contained a single animal protagonist. The videos were edited to exclude other people or animals apart from the sole target animal in an attempt to exclude any indicators of a CS relationship between the animal and other subjects. Similarly, participants were asked to mute the sound on their computers because some of the videos featured auditory communication that was indicative of communal relationships (e.g., a pet owner speaking to her dog in a very affectionate way). This was done so that the videos only differed on the independent variable of cuteness.

**Supplementary Table 10.** Mean cuteness scores of pretest videos in Study 1

| Video | Mean cuteness score | Video link^[[1]](#footnote-1)^ | Timeframe |
| --- | --- | --- | --- |
| **Experimental/Cute** | | | |
| **Video 1** | **5.75** | <https://www.youtube.com/watch?v=8HVWitAW-Qg> | 0:55 – 1:19 |
| **Video 2** | **5.50** | <https://www.youtube.com/watch?v=_v94XqFW4Qw> | 0:04 – 0:37 |
| **Video 3** | **5.25** | <https://www.youtube.com/watch?v=JlWlnBWVQLE> | 0:34 – 1:13 |
| **Video 4** | **5.25** | <https://www.youtube.com/watch?v=15XN60jQ3e0> | 0:22 – 0:57 |
| Video 5 | 4.50 | <https://www.youtube.com/watch?v=-d_hu0O_ww4> | 0:17 – 1:05 |
| Video 6 | 4.50 | <https://www.youtube.com/watch?v=hhoQqN9oUpo> | 4:27 – 4:52 |
| Video 7 | 4.25 | <https://www.youtube.com/watch?v=nDyu-z8q7ko> | 1:59 – 2:44 |
| Video 8 | 3.75 | <https://www.youtube.com/watch?v=amtuB-2wGeQ> | 0:02 – 0:33 |
| **Control/non-cute** | | | |
| **Video 1** | **1.25** | <https://www.youtube.com/watch?v=jZuUGJRtre> | 0:45 – 1:14 |
| **Video 2** | **1.50** | <https://www.youtube.com/watch?v=VqPMP9X-89o> | 1:24 – 1:57 |
| **Video 3** | **1.75** | <https://www.youtube.com/watch?v=H8oQBYw6xxc> | 1:18 – 1:52 |
| **Video 4** | **2.00** | <https://www.youtube.com/watch?v=c1C9rM76BpI> | 1:20 – 1:44 |
| Video 5 | 2.50 | <https://www.youtube.com/watch?v=b3w9ZbRQIek> | 3:35 – 3:54 |
| Video 6 | 2.50 | <https://www.youtube.com/watch?v=57wyRdd1gj0> | 13:10 – 13:39 |
| Video 7 | 2.75 | <https://www.youtube.com/watch?v=VTV23B5gBsQ> | 0:05 – 0:33 |
| Video 8 | 3.25 | <https://www.youtube.com/watch?v=5ckHs6rEBJE> | 2:17 – 2:42 |

Note. Four videos in each condition with the highest (experimental group) and lowest (control group) ranking on cuteness are outlined in bold. These eight videos were selected as stimuli for study 1. Timeframe indicates the start and the end of the episode shown (min:sec).

## Study 1 stimuli

Below you can see one screenshot each of one of the videos in each condition for Study 1.


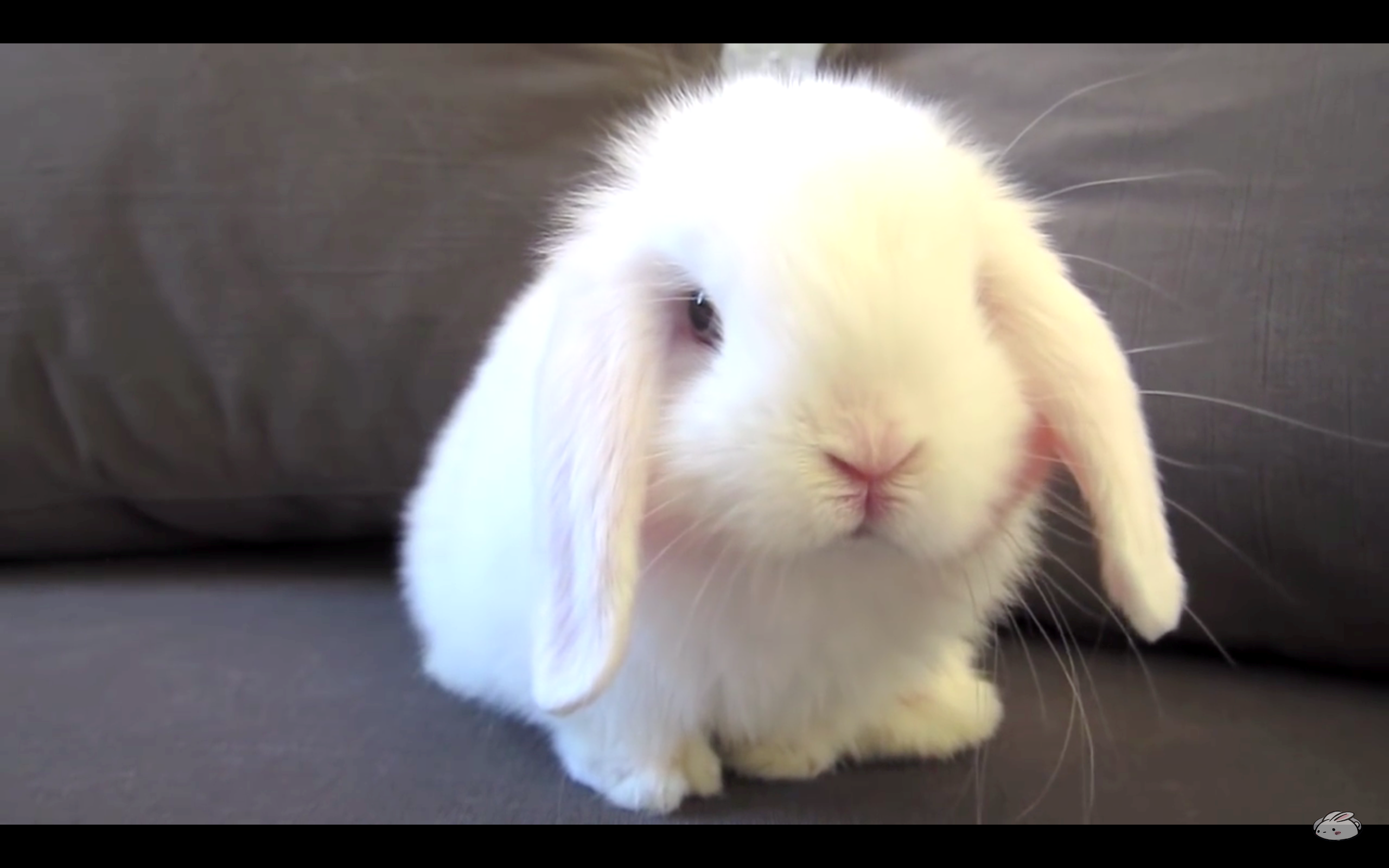


**Supplementary Figure 1.** Screenshot of video in high cuteness condition


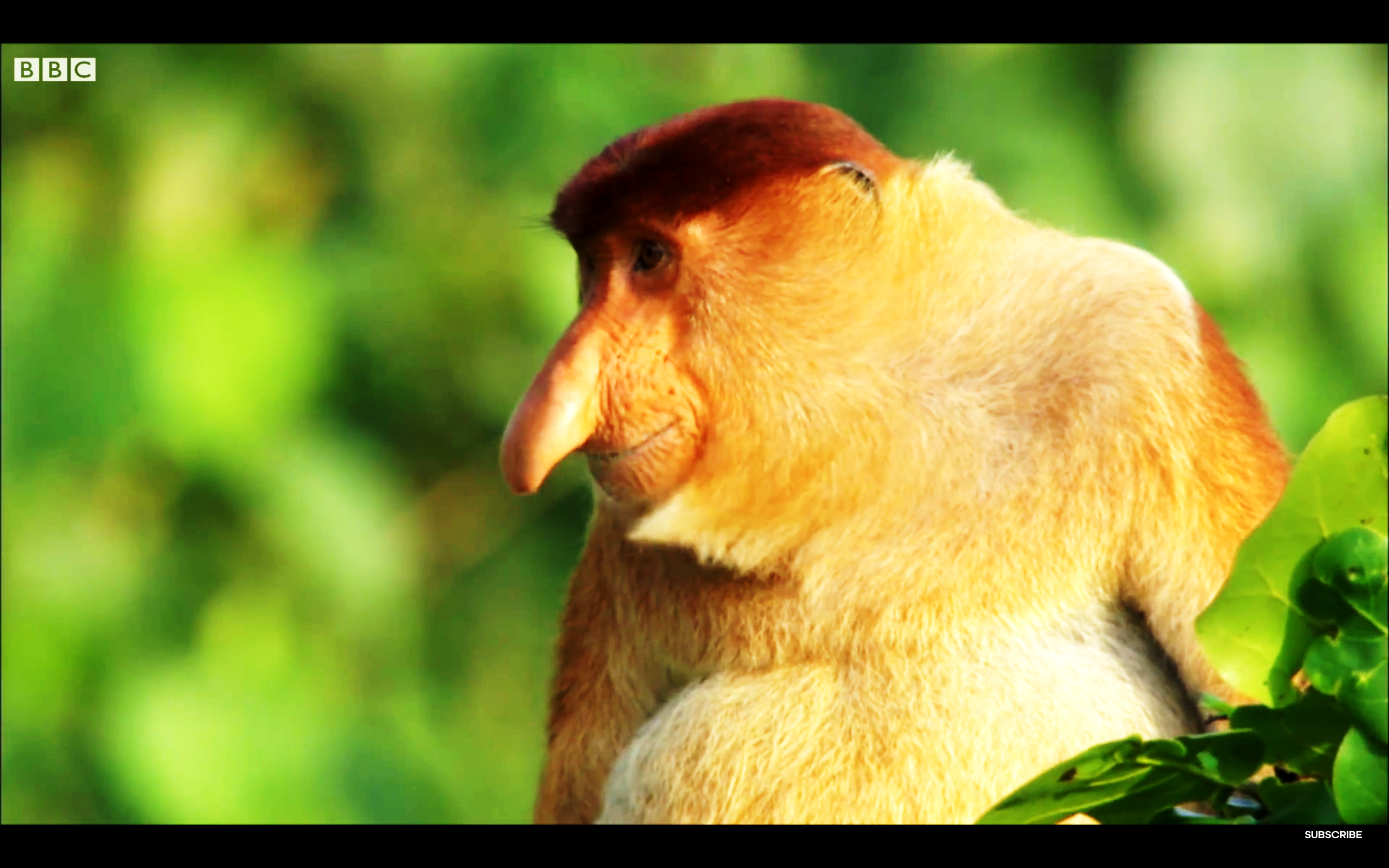


**Supplementary Figure 2.** Screenshot of video in low cuteness condition

## Study 2 stimuli

Below we provide links to the stimuli used in Study 2, which we uploaded to Youtube, and example screenshots from one of the videos in each condition.

**Supplementary Table 11.** Stimuli used in Study 2

| Protagonists | Video link | Length in min |
| --- | --- | --- |
| High CS condition | | |
| Kitten and human | <https://www.youtube.com/watch?v=ngxg-ZhojVY> | 0:46 |
| Two kittens | <https://www.youtube.com/watch?v=qbnYWZKwgMk> | 0:47 |
| Two puppies | <https://www.youtube.com/watch?v=JfXMMoVQHHs> | 0:25 |
| Puppy and mother | <https://www.youtube.com/watch?v=pBUxVELko10> | 0:44 |
| Low CS condition | | |
| Kitten and human | <https://www.youtube.com/watch?v=_RCqVy3TPkw> | 0:33 |
| Two kittens | <https://www.youtube.com/watch?v=3w_gVmSEI2w> | 0:44 |
| Two puppies | <https://www.youtube.com/watch?v=gvJqMQy8P2M> | 0:30 |
| Puppy and mother | <https://www.youtube.com/watch?v=zeln7wS5wPY> | 0:27 |


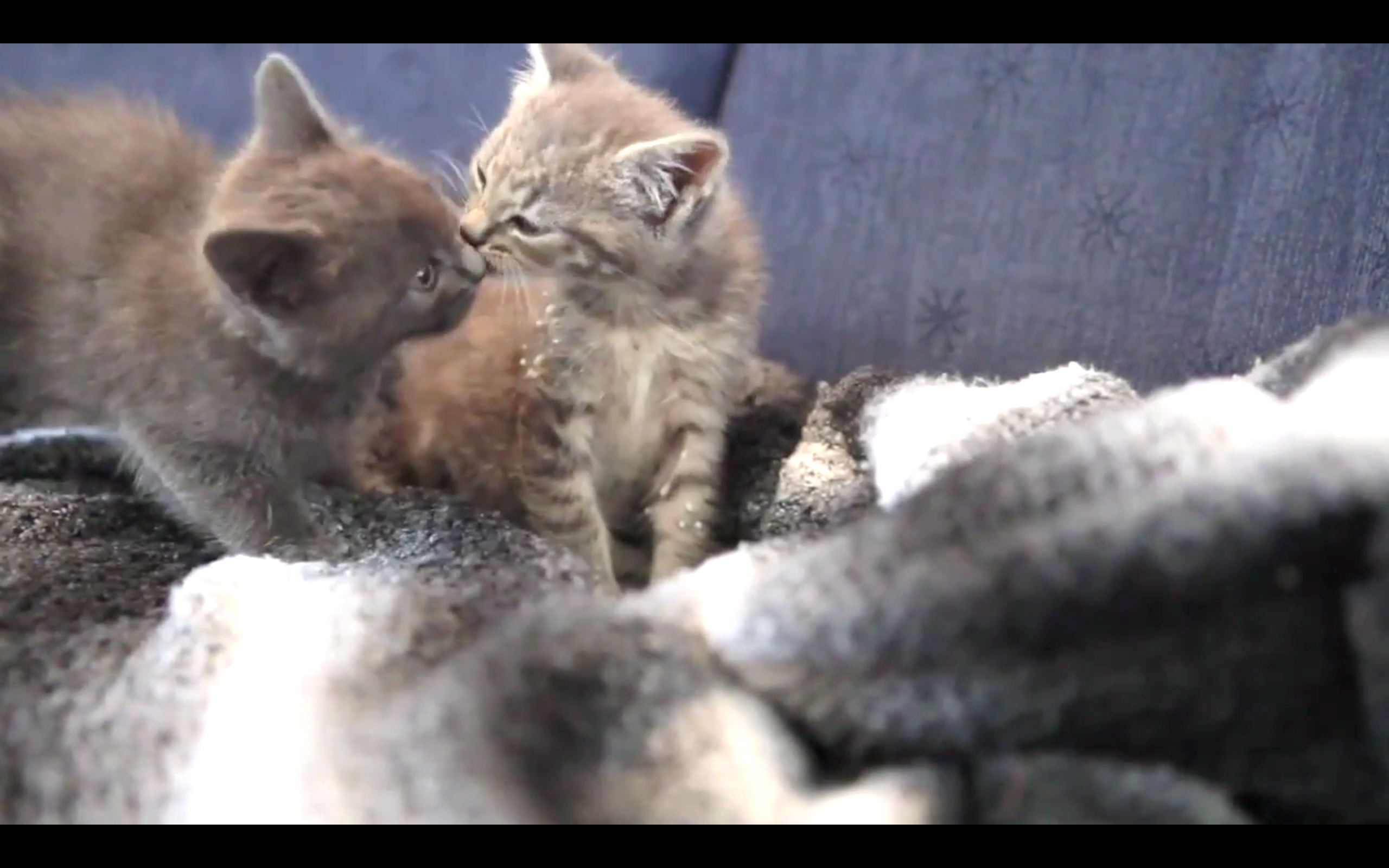


**Supplementary Figure 3.** Screenshot of video in high communal sharing condition


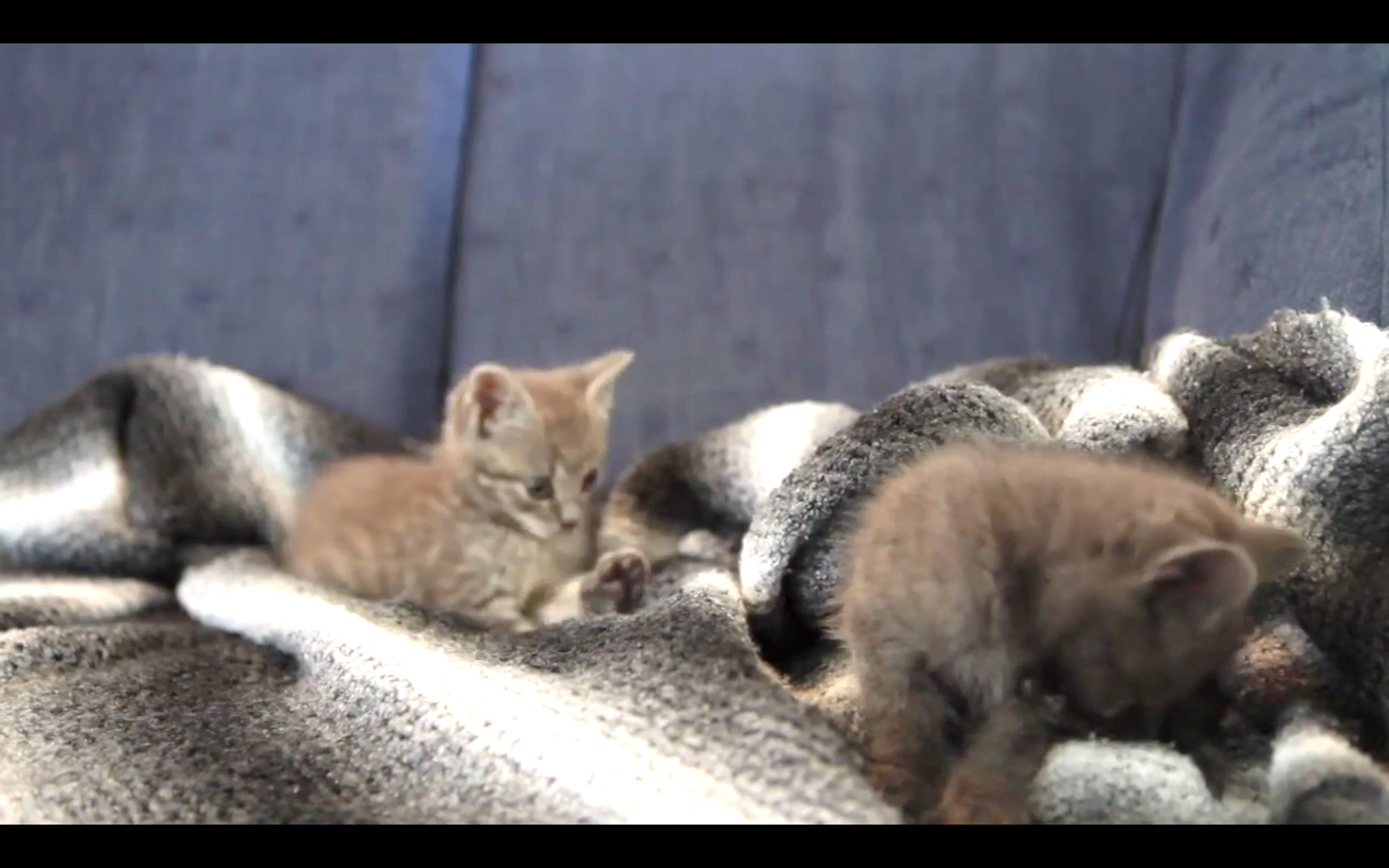


**Supplementary Figure 4.** Screenshot of video in low communal sharing condition

# Supplementary analyses and results

## Study 1

**Demographic information.** Of our final sample *N* = 165 had no children, *N* = 16 had one child, *N* = 19 had two children, *N* = 15 had more than two children, *N* = 2 answers were missing, and *N* = 107 had a pet (*N* = 2 missing).

**Factor analysis of cuteness scale.** The data collected from the 9-item cuteness scale, excluding distractor items (control condition: α =.82, cute condition: α =.86), were subjected to a factor analysis with oblimin rotation. Factor extraction was based on a parallel analysis suggesting a two-factor solution (Supplementary Table A). Six positively worded cuteness items formed factor 1 (Eigenvalue: 5.35), while three reverse-coded items 2 (Eigenvalue: 1.30), respectively. This model explained a total of 74% of the score variance (factor 1: 59%, factor 2: 14%). The Eigenvalue of factor 1 (6.00) and factor 2 (.42) were above the randomly generated value from the parallel analysis for factor 1 (.51) and 2 (.16) and was therefore kept. Factor 3 was discarded due to its factor analysis Eigenvalue (.06) being below its parallel analysis value (.11). A reliability analysis of the 9-item scale was performed. Corrected total item correlation found that the six cuteness items (‘It is adorable’ = .864, ‘It is sweet’ = .786, ‘It looks so soft’ = .768, ‘It is cute’ = .871, ‘It is cuddly’ = .815, ‘I would like to pet it’ = .763) and the three reversed items (‘It is repulsive’ = .129, ‘It is huge’ = -.020, ‘It looks really old’ = .105) formed two orthogonal variates. Because these items loaded on a separate factor, the 3 reverse-coded items were excluded from the scale, leaving a six-item cuteness scale measuring only one factor (α =.98).

**Supplementary Table 12.** Three extracted factors from an exploratory factor analysis of the cuteness scale (9 items) as well as each item’s communalities and factor loading on all three factors.

| Indicators | Factor loadings | |
| --- | --- | --- |
|  | Factor 1 | Factor 2 |
| ***Factor 1: Cuteness items*** |  |  |
| It is adorable | **.960** | -.006 |
| It is sweet | **.976** | -.038 |
| It looks so soft | **.861** | .055 |
| It is cute | **.928** | .043 |
| It is cuddly | **.942** | .001 |
| I would like to pet it | **.925** | -.029 |
| ***Factor 2: Reversed items*** |  |  |
| It is repulsive | .050 | **.700** |
| It is huge | .236 | **.419** |
| It looks really old | -.054 | **.744** |

**Differences in Cuteness Responses with Regard to Demographic Indicators.** We also tested whether cuteness responses differed across gender, individuals having a pet, and individuals having a child. In a mixed model we added the condition, gender, pet, number of children mean centered and the two-way interactions with our demographic indicators and condition. The cuteness score was used as the dependent variable. Results are displayed in Supplementary Table 13. There was no statistically significant main effect for gender as males (*M*=2.77, *SD*=2.21) or females (*M*=2.91, *SD*=2.38) did not differ in their cuteness responses. However, we found a main effect for ownership of a pet. Participants owning a pet showed on average stronger cuteness responses (*M*=3.05, *SD*=2.34) in contrast to participants without a pet (*M*=2.66, *SD*=2.26). In addition, we found that these two main effects were moderated by condition. The effect of pet ownership was stronger for the cute videos (Pet: *M*=5.08, *SD*=1.06; No Pet: *M*=4.43, *SD*=1.58) than for the control videos (Pet: *M*=1.01, *SD*=1.22; No Pet: *M*=.90, *SD*=1.21). The effect of gender was also stronger in the cute condition (Male: *M*=4.46, *SD*=1.56; Female: *M*=4.98, *SD*=1.18) in contrast to the control condition (Male: *M*=1.09, *SD*=1.28; Female: *M*=.85, *SD*=1.16). In fact, the effect was slightly reversed for the control videos. Finally, we found no main effect or interaction including the number of children.

**Supplementary Table 13.** Study 1: Prediction of Cuteness by Condition, Gender, Pet, Child, and their Two-Way interactions using Mixed Models

| **Predictor** | **F** | **df 1, df 2** | **p** | **B [95% CI]** | **d (r*)** |
| --- | --- | --- | --- | --- | --- |
| **Condition** | 94.96 | 1,34 | <.001 | 5.48 [4.42, .6.54] | - |
| **Gender** | 1.13 | 1,206 | .288 | -.14 [-.39, .12] | -.06 |
| **Pet** | 8.72 | 1,207 | .004 | -.37 [-.62, -.13] | -.16 |
| **Child** | .002 | 1,209 | .963 | -.003 [-.14, .13] | -.05* |
| **Condition*Gender** | 8.68 | 1,207 | .004 | -.66 [-1.11, -.23] | - |
| **Condition*Pet** | 4.78 | 1,208 | .03 | -.49 [-.92, -.05] | - |
| **Condition*Child** | .17 | 1,210 | .681 | -.05 [-.29, .19] | - |

*Note.* All the outcome variable was measured on scales from 0 to 6. All factors were contrast coded (condition: -.5 – control; .5 – cute) and the covariate (Child) was measured on a scale from 1 to 4 and mean centered. For all models intercepts were allowed to vary randomly across participants and video. Values denoted with * represent correlation coefficients.

## Study 2

**Additional demographics.** Of our final sample *N* = 123 had no children, *N* = 8 had one child, *N* = 3 had two children, *N* = 3 had more than two children (*N* = 2 did not indicate number of children), and *N* = 52 had a pet (*N* = 1 did not indicate whether they had a pet).

### 3.2.1 Additional Analyses Using Separate Sign Scores.

**Supplementary Table 14.** Study 1: Prediction of Tears, Chills, Warmth, Choked Up and Exhilaration by Condition, Order, Empathic Concern (EC) and their Two-Way Interactions using Mixed Models

| Predictor | F | df1,df2 | *p* | B [95% CI] | *d* (*r**) |
| --- | --- | --- | --- | --- | --- |
|  | **Tears (Model 1)** | | | | |
| Condition | 16.94 | 1,212 | < .001 | .21 [.10, .31] | .31 |
| Order | .006 | 1,212 | .939 | .003 [-.09, .10] | .006 |
| EC | .68 | 1,213 | .408 | .04 [-.05, .12] | .05* |
| Condition*Order | 2.99 | 1,213 | .08 | .27 [-.03, .57] | - |
| Condition*EC | 5.32 | 1,212 | .02 | .13 [.02, .23] | - |
| Order*EC | .68 | 1,212 | .409 | .05 [-.06, .15] | - |
|  | **Chills (Model 2)** | | | | |
| Condition | 1.76 | 1,5 | .243 | -.19 [-.46, .09] | -.22 |
| Order | 1.57 | 1,208 | .212 | -.09 [-.23, .05] | -.10 |
| EC | .38 | 1,213 | .540 | .03 [-.07, .13] | .03* |
| Condition*Order | 1.93 | 1,213 | .166 | .26 [-.10, .63] | - |
| Condition*EC | .16 | 1,208 | .686 | .03 [-.12, .18] | - |
| Order*EC | .81 | 1,208 | .370 | .07 [-.09, .22] | - |
|  | **Warmth (Model 3)** | | | | |
| Condition | 125.48 | 1,212 | <.001 | .97 [.79, .1.15] | .85 |
| Order | 1.32 | 1,212 | .251 | -.10 [-.27, .07] | -.08 |
| EC | 10.02 | 1,213 | .002 | .22 [.08, .36] | .16* |
| Condition*Order | 2.03 | 1,213 | .16 | .36 [-.13, .86] | - |
| Condition*EC | 16.45 | 1,212 | < .001 | .38 [.20, .57] | - |
| Order*EC | .61 | 1,212 | .434 | .07 [-.11, .26] | - |
|  | **Choked Up (Model 4)** | | | | |
| Condition | 2.25 | 1,3 | .219 | .09 [-.03, .22] | .14 |
| Order | .02 | 1,210 | .886 | -.01 [-.12, .10] | -.01 |
| EC | .19 | 1,212 | .666 | .02 [-.06, .10] | .02* |
| Condition*Order | 2.12 | 1,212 | .147 | .22 [-.07, .51] |  |
| Condition*EC | 4.11 | 1,211 | .04 | .12 [.01, .24] |  |
| Order*EC | 2.92 | 1,210 | .09 | .10 [-.02, .22] |  |
|  | **Exhilaration (Model 5)** | | | | |
| Condition | 68.10 | 1,4 | < .001 | 1.22 [.93, 1.52] | .76 |
| Order | 1.02 | 1,203 | .314 | -.12 [-.35, .11] | -.07 |
| EC | 10.46 | 1,209 | .001 | .32 [.13, .52] | .17* |
| Condition*Order | 2.78 | 1,210 | .09 | .61 [-.10, 1.32] | - |
| Condition*EC | 9.33 | 1,205 | .003 | .39 [.13, .64] | - |
| Order*EC | .26 | 1,203 | .608 | .07 [-.18, .32] | - |

*Note.* All outcome variables were measured on scales from 0 to 6. All factors were contrast coded (condition: -.5 – control; .5 – cute, order: -.5 – first; .5 - second) and the covariate (EC) was measured on a scale from 1 to 5 and mean centered. For all models intercepts were allowed to vary randomly across participants and video. Values denoted with * represent correlation coefficients.

### Non-parametric models for main analysis.

**Supplementary Table 15.** Wilcoxon signed rank test with condition as predictor for each of the dependent variables.

|  | **Median** | | **Z** | ***p*** | ***r**** |
| --- | --- | --- | --- | --- | --- |
| **Variable** | Non-Cute | Cute |  |  |  |
| Cuteness | .33 | 5 | -12.57 | <.001 | -.60 |
| Labels | 0 | 1.67 | -10.77 | <.001 | -.52 |
| Signs | .17 | .75 | -9.74 | <.001 | -.47 |
| Motivation | 0 | .5 | -8.51 | <.001 | -.41 |
| Positive Valence | 2 | 4 | -10.35 | <.001 | -.50 |

*the effect size (*r*) is calculated based on Pallant (2007): *z* / √*N.* Number of cases (*N*) = 434.

### Additional Analyses Using Separate Sign Scores.

**Supplementary Table 16.** Study 2: Prediction of Communal Sharing, Cuteness, Labels, and Signs by Condition, Animal Type, Order, Video Version, Empathic Concern (EC) and the Interactions Condition x Order, and Animal Type x Version using Mixed Models

| Predictor | F | df1,df2 | *p* | B [95% CI] | *d* (*r**) |
| --- | --- | --- | --- | --- | --- |
|  | **Tears (Model 1)** | | | |  |
| Condition | 4.49 | 1,133 | .04 | .17 [.01, .33] | .21 |
| Animal Type | .53 | 1,132 | .466 | .06 [-.10, .22] | .07 |
| Order | .15 | 1,133 | .699 | -.03 [-.19, .13] | -.04 |
| Video Version | .27 | 1,252 | .605 | .05 [-.14, .23] | .06 |
| Condition*Order | 1.22 | 1,137 | .272 | .25 [-.19, .69] | - |
| Animal Type*Version | .87 | 1,230 | .351 | .17 [-.19, .53] | - |
|  | **Chills (Model 2)** | | | |  |
| Condition | 6.10 | 1,118 | .01 | .21 [.04, .37] | .27 |
| Animal Type | .33 | 1,118 | .565 | .05 [-.11, .21] | .06 |
| Order | 4.86 | 1,118 | .03 | -.18 [-.35, -.02] | -.24 |
| Video Version | .07 | 1,267 | .786 | -.02 [-.20, .16] | -.03 |
| Condition*Order | .61 | 1,121 | .438 | -.15 [-.53, .23] | - |
| Animal Type*Version | .45 | 1,251 | .504 | .12 [-.23, .47] | - |
|  | **Warmth (Model 3)** | | | |  |
| Condition | 13.58 | 1,130 | < .001 | .53 [.25, .80] | .29 |
| Animal Type | 1.37 | 1,130 | .245 | -.17 [-.44, .11] | -.09 |
| Order | 5.52 | 1,130 | .02 | -.34 [-.61, -.06] | -.19 |
| Video Version | 1.55 | 1,210 | .215 | -.23 [-.58, .13] | -.13 |
| Condition*Order | .83 | 1,135 | .365 | -.49 [-1.53, .56] | - |
| Animal Type*Version | .22 | 1,190 | .643 | -.16 [-.84, .52] | - |
|  | **Choked Up (Model 4)** | | | |  |
| Condition | 1.48 | 1,133 | .227 | .10 [-.06, .26] | .13 |
| Animal Type | 1.59 | 1,132 | .210 | .10 [-.06, .26] | .13 |
| Order | 1.50 | 1,133 | .222 | -.10 [-.26, .06] | -.13 |
| Video Version | .04 | 1,264 | .835 | .02 [-.16, .20] | -.02 |
| Condition*Order | .20 | 1,135 | .659 | -.10 [-.49, .31] | - |
| Animal Type*Version | 1.66 | 1,245 | .199 | .23 [-.12, .58] | - |
|  | **Exhilaration (Model 5)** | | | |  |
| Condition | 15.86 | 1,135 | < .001 | .56 [.29, .83] | .35 |
| Animal Type | 1.57 | 1,134 | .212 | -.18 [-.45, .10] | -.11 |
| Order | 3.94 | 1,135 | .049 | -.28 [-.55, -.01] | -.17 |
| Video Version | 4.58 | 1,237 | .03 | -.37 [-.70, -.03] | -.23 |
| Condition*Order | 2.26 | 1,137 | .135 | -.68 [-1.56, .20] | - |
| Animal Type*Version | .36 | 1,215 | .548 | .20 [-.45, .85] | - |

### Non-parametric models of main analyses.

**Supplementary Table 17.** Wilcoxon signed rank test with condition as predictor for each of the dependent variables.

|  | **Median** | | **Z** | ***p*** | ***r**** |
| --- | --- | --- | --- | --- | --- |
| **Variable** | low CS | high CS |  |  |  |
| Appraisal | .75 | 3 | -7.98 | <.001 | -.48 |
| Cuteness | 4.33 | 5 | -6.65 | <.001 | -.40 |
| Labels | 1 | 2 | -5.02 | <.001 | -.30 |
| Signs | .42 | .58 | -3.38 | <.001 | -.20 |
| Humanization | 2 | 3 | -1.76 | .039 | -.11 |
| Motivation | .75 | 1.5 | -3.72 | <.001 | -.22 |
| Positive Valence | 3 | 5 | -5.34 | <.001 | -.32 |

*the effect size (*r*) is calculated based on Pallant (2007): *z* / √*N*. Number of cases (*N*) = 278.

1. Note the possibility that some of the video links may have gone dead in the time after this research has been published, depending on the individual uploaders of the videos. For this reason, we provide screenshots of one video from each condition. [↑](#footnote-ref-1)
